# Tankyrase-1-mediated degradation of Golgin45 regulates glycosyltransferase trafficking and protein glycosylation in Rab2-GTP-dependent manner

**DOI:** 10.1038/s42003-021-02899-0

**Published:** 2021-12-07

**Authors:** Xihua Yue, Neeraj Tiwari, Lianhui Zhu, Hai Dang Truong Ngo, Jae-Min Lim, Bopil Gim, Shuaiyang Jing, Yijing Wang, Yi Qian, Intaek Lee

**Affiliations:** 1 School of Life Science and Technology, ShanghaiTech University, Shanghai, China; 2 Department of Cell Biology, Yale University School of Medicine, New Haven, CT 06520 USA; 3 Department of Chemistry, Changwon National University, Changwon, Gyeongnam South Korea; 4 School of Physical Science and Technology, ShanghaiTech University, Shanghai, China; 5 University of Chinese Academy of Sciences, Beijing, China

**Keywords:** Golgi, Golgi

## Abstract

Altered glycosylation plays an important role during development and is also a hallmark of increased tumorigenicity and metastatic potentials of several cancers. We report here that Tankyrase-1 (TNKS1) controls protein glycosylation by Poly-ADP-ribosylation (PARylation) of a Golgi structural protein, Golgin45, at the Golgi. TNKS1 is a Golgi-localized peripheral membrane protein that plays various roles throughout the cell, ranging from telomere maintenance to Glut4 trafficking. Our study indicates that TNKS1 localization to the Golgi apparatus is mediated by Golgin45. TNKS1-dependent control of Golgin45 protein stability influences protein glycosylation, as shown by Glycomic analysis. Further, FRAP experiments indicated that Golgin45 protein level modulates Golgi glycosyltransferease trafficking in Rab2-GTP-dependent manner. Taken together, these results suggest that TNKS1-dependent regulation of Golgin45 may provide a molecular underpinning for altered glycosylation at the Golgi during development or oncogenic transformation.

## Abstract (web summary)

Xihua Yue et al. report that Tankyrase-1 controls poly-ADP-ribosylation of Golgin45, a Golgi structural protein, which has a profound influence on glycosyltransferase trafficking and protein glycosylation at the Golgi. This may shed new light onto mechanisms involved in development and oncogenic transformation.

## Introduction

Tankyrase1 (TNKS1) is a peripheral membrane protein with diverse roles, ranging from telomere maintenance and GLUT4 vesicle trafficking to the regulation of Wnt/β-catenin signaling for the control of cellular differentiation during embryonic development and tissue homeostasis in adult tissues^1–6^. During interphase, TNKS1 is mainly localized to the Golgi apparatus, although it had been unknown how TNKS1 associates with the Golgi membranes^2^. Zhang et al^7^ identified axin1 and BLZF1 (also known as Golgin45 or JEM-1) as the two major targets for TNKS1-dependent poly-ADP-ribosylation (PARylation), leading to their rapid ubiquitination and proteasome-dependent degradation. While TNKS1-dependent PARylation of axin1 was shown to positively regulate Wnt-signaling pathway, the role of Golgin45 PARylation has remained unknown.

On the other hand, Golgin45 is a Golgi structural protein that has been shown to directly interact with at least four Golgi-associated proteins, including ACBD3, GRASP55, Syntaxin5 and a small GTPase, Rab2-GTP^8–10^. Golgin45 is also called BLZF1, Basic Leucine Zipper Nuclear Factor 1, as it is known to function as a nuclear cofactor^11^. ACBD3 recruits TBC1D22, a putative Rab33b-GTPase activating protein (GAP), to the large multi-protein complex, potentially leading to a unique membrane micro-domain organization at the cis- and *medial*-Golgi^9^. In addition, we have recently shown that Golgin45-Syntaxin5 interaction contributes to the structural integrity of the Golgi stack by inhibiting intercisternal fusion between adjacent Golgi cisterna^10^.

In this study, we aimed to investigate the physiological role of TNKS1-dependent degradation of Golgin45. We report here that TNKS1 association with the Golgi membranes is mediated by Golgin45. TNKS1 and GRASP55 bind Golgin45 in a mutually exclusive manner to form distinct protein complexes. Four of the five ARC domains of TNKS1 directly bind ^18^RGAGDG^23^ motif, located in Golgin45 N-terminus, and this interaction seems partly regulated via phosphorylation of Golgin45 N-terminal domain by a cell cycle-dependent kinase, Cdc2. Importantly, FRAP of β(1, 4)-galactosyltransferase fused to GFP (GalT1-GFP), a Golgi glycosyltransferease, and Glycomic analysis by mass spectrometry indicated that Golgin45 protein stability drastically influences protein glycosylation by regulating glycosyltransferase trafficking dynamics in a Rab2-GTP-dependent manner. These results suggest that TNKS1 activity is closely associated with modulation of Golgi function to a previously unanticipated degree and may greatly influence protein glycosylation at the Golgi during development or oncogenic transformation.

## Results

**Fig. 1:**
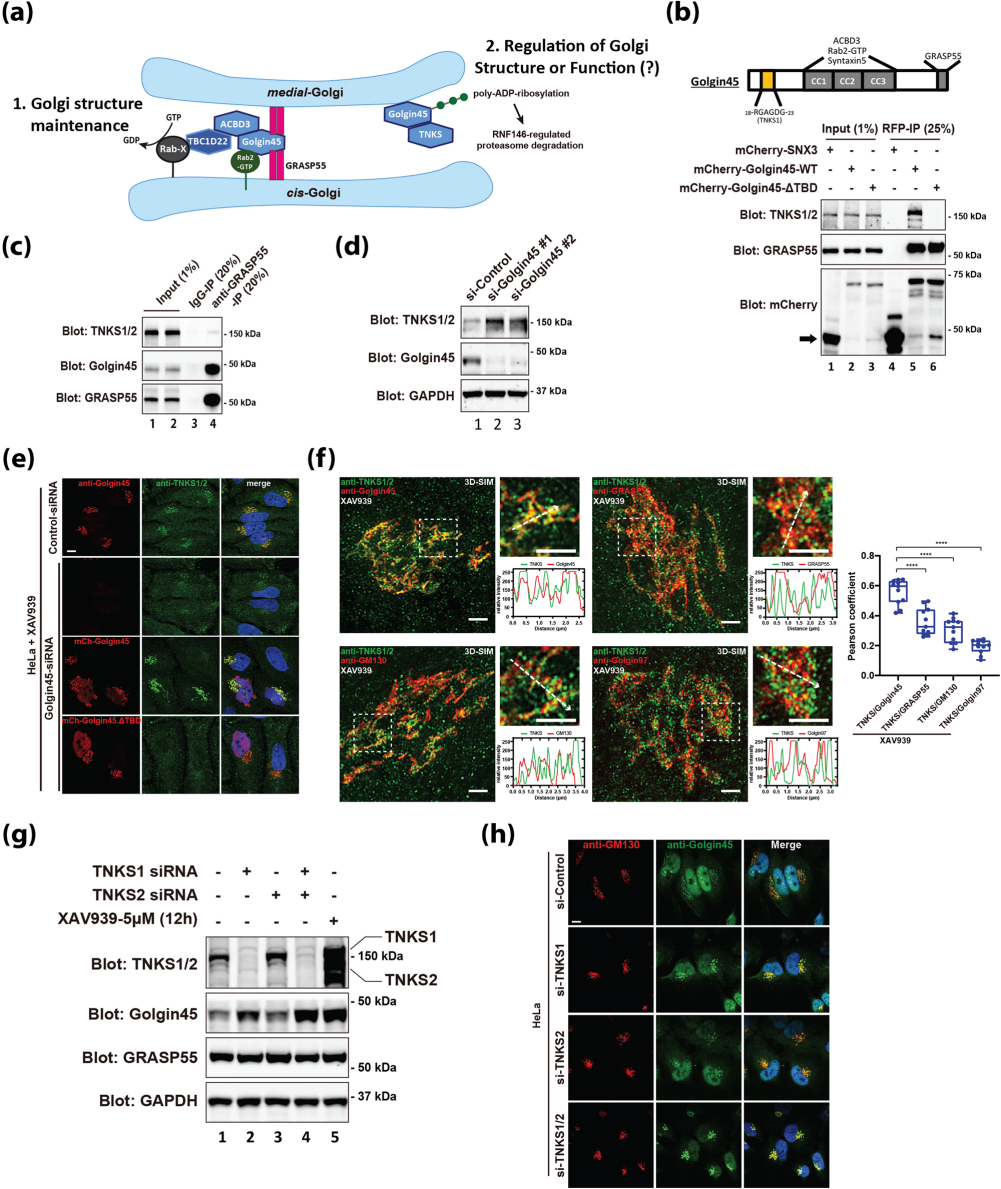
TNKS1 targeting to the Golgi membranes is mediated by Golgin45 and localizes to the cis- and medial-Golgi cisternae. **a** A brief overview of Golgin45 protein complexes. Golgin45 forms a multi-protein complex with GRASP55 and ACBD3 that contributes to Golgi structure maintenance. Golgin45 was identified as a substrate for Tankyrase1-dependent PARylation and subsequent proteasomal degradation. The physiological role of TNKS1–Golgin45 interaction is currently unknown. **b** Golgin45 binding to TNKS1 is dependent on Tankyrase-binding domain (TBD). The protein extracts from HeLa cells transfected with mCherry tagged Sorting Nexin 3 (mCherry-SNX3 as a control), mCherry-Golgin45 or mCherry-Golgin45-ΔTBD (deletion of TBD at the Golgin45 N-terminal domain) were immunoprecipitated with anti-RFP agarose beads. These lysates and the immunoprecipitates (anti-RFP IPs) were analyzed by western blotting using anti-mCherry antibody and antibodies against the indicated proteins. Deletion of TBD at the Golgin45 N-terminal domain abrogates its interaction with endogenous TNKS1. Experiments were repeated three times and representative western blots are shown here. **c** Immunoprecipitation experiments showing that endogenous GRASP55 selectively forms a complex with Golgin45, but not with TNKS1/2, suggesting that TNKS1/2 are excluded from GRASP55-Golgin45 complex. The protein extracts from HeLa cells were immunoprecipitated with anti-GRASP55 or normal rabbit IgG. These lysates and the immunoprecipitates were analyzed by western blotting using antibodies against the indicated proteins. Representative blots are shown and experiments were repeated three times. **d** Golgin45 depletion results in increased TNKS1 protein level in HeLa cells. HeLa cells were transfected with two independently Golgin45 siRNAs for 72 h, followed by lysis and western blot analysis. Representative blots are shown and experiments were repeated three times. **e** Golgi localization of TNKS1/2 is mediated by Golgin45. Confocal micrographs of HeLa cells showed that Golgin45 knockdown distrupts the Golgi localization of TNKS1/2, which could be restored by exogenous expression of RNAi-resistant mCherry-Golgin45 other than mCherry-Golgin45-ΔTBD. HeLa cells were transfected with Golgin45 siRNA for 48 h, followed by XAV939 treatment and exogenous expression of RNAi-resistant mCherry-Golgin45 or mCherry-Golgin45-ΔTBD overnight. Scale bar, 10 µm. **f** Super-resolution 3D-SIM images showing a high degree of co-localization between TNKS1/2 and Golgin45 and lower co-localization between TNKS1/2 and GM130 (*cis*-Golgi marker) or GRASP55 (*medial*-Golgi marker) or Golgin97 (*TGN* marker). Line profiles through regions of interest were analyzed by Fiji. Scale bars, 2 μm. Co-localization (Pearson’s R) was determined and subjected to two-tailed, unpaired t test (*n* = 10 cells/combination, mean ± SD, ****, *p* < 0.0001). **g**, **h** Effects of knockdown of TNKS1 or/and TNKS2 on the protein level of Golgin45. HeLa cells were transfected with TNKS RNAi oligoes for 72 h, followed by lysis and western blot analysis (**g**), or cells were stained with antibodies against GM130 and Golgin45. Scale bar, 10 µm (**h**). HeLa cells treated with XAV939 were shown as a positive control for western blot. Knocking down TNKS1 but not TNKS2 increased the protein level and Golgi intensity of Golgin45. Simultaneous depletion of both TNKS1 and TNKS2 resulted in maximal increase of Golgin45 protein level and Golgi intensity, compared to TNKS1 depletion alone. Representative images/blots are shown and experiments were repeated three times.

Golgin45 forms a multi-protein complex with GRASP55 and ACBD3 that contributes to Golgi structure maintenance, but the physiological role of its newly identified interaction with TNKS1 at the Golgi and subsequent degradation by the proteasome has remained unknown, as illustrated in Fig. 1a.

### TNKS1 forms a distinct protein complex with Golgin45 in a GRASP55-independent manner

Prior to an in-depth study of its functional role at the Golgi, we first confirmed whether TNKS1 can directly interact with Golgin45^7^. To this end, we performed co-immunoprecipitation experiments, using mCherry-Golgin45 and mCherry-Golgin45-ΔTBD (Tankyrase Binding Domain: ^18^RGAGDG^23^) deletion mutant. HeLa cells were transfected with either of these two constructs or mCherry-SNX3 (Sorting Nexin-3) as a negative control for 18 h, followed by cell lysis and immunoprecipitation using anti-RFP agarose beads.

The results showed that wild-type Golgin45 pulled down both endogenous GRASP55 and TNKS1, whereas TNKS1 binding to mCherry-Golgin45-ΔTBD was selectively disrupted (Fig. 1b), confirming the binding specificity between the two proteins. As expected, mCherry-SNX3 failed to pull down either GRASP55 or TNKS1.

Golgin45 is known to form a tight complex with GRASP55 and contributes to Golgi structure maintenance^8,12^. Thus, we next studied whether TNKS1 can associate with Golgin45-GRASP55 complex by performing co-immunoprecipitation experiments using an antibody against endogenous GRASP55 and blotting for endogenous Golgin45 and TNKS1. Surprisingly, GRASP55 failed to pull down TNKS1, although the immunoprecipitated complex contained a significant amount of Golgin45 (Fig. 1c). Taken together, these results suggested that TNKS1 and GRASP55 may independently interact with Golgin45 in a mutually exclusive manner.

While testing RNAi oligos for Golgin45 knockdown, we found that Golgin45 depletion results in increased TNKS1 protein level in HeLa cells. As shown in Fig. 1d, there was a significant increase in TNKS1 protein level in cells treated with two different RNAi oligos against Golgin45, strongly indicating that TNKS1 protein stability during interphase may be heavily dependent on its PARylation activity on Golgin45 and subsequent auto-PARylation^7^.

### TNKS1 targeting to the Golgi is mediated by Golgin45 and localizes to the cis- and medial-Golgi cisternae

Although it has been well known that TNKS1 localizes to the Golgi during interphase, the mechanism by which TNKS1 is targeted to the Golgi has been unknown^2^. Because Golgin45 was reported to be PARsylated and degraded in TNKS1-dependent manner^7^, we then asked whether TNKS1–Golgin45 interaction is required for TNKS1 targeting to the Golgi.

While screening for a good commercial TNKS1 antibody for indirect immunofluorescent staining experiments, we found that only one commercially available TNKS1 antibody was suitable for indirect immunofluorescent staining. Unexpectedly, this antibody showed strong Golgi staining only when the cells were treated with XAV939, a well-known chemical inhibitor of TNKS1^13^ (supplementary Fig. 1a), suggesting that auto-PARylation/degradation of TNKS1 is quite high under physiological condition. This finding led us to XAV939 pre-treatment as a standard protocol for all TNKS1 immunofluorescent staining experiments in this study.

In order to investigate whether Golgin45 may function as a docking factor for TNKS1 association with the Golgi membranes, HeLa cells transfected with Golgin45 RNAi or a control scrambled RNAi for 72 h were pretreated with 5 μM XAV939, followed by staining with antibodies against TNKS1/2 and Golgin45.

The confocal results showed that TNKS association with the Golgi was completely lost upon Golgin45 depletion, which could be restored by re-expression of RNAi-resistant mCherry-Golgin45 WT (Fig. 1e). On the other hand, expression of mCherry-Golgin45-ΔTBD failed to restore Golgi localization of TNKS1, suggesting that TNKS1 association with the Golgi membranes is clearly mediated by a direct interaction between Golgin45 and TNKS1 via the TBD of Golgin45 (^18^RGAGDG^23^). These results were also reproduced well using COS7 cells (Supplementary Fig. 1b), indicating that Golgin45-dependent targeting of TNKS1 to the Golgi is likely to be conserved across different species or cell types.

Because both Golgin45 and its binding partner, GRASP55, are found in the *cis*- and *medial*-Golgi cisternae^8^, these results raised a possibility that TNKS1 may similarly localize to the *cis*- and *medial*-Golgi compartments like Golgin45/GRASP55 complex.

To test this, we stained HeLa cells using antibodies against GM130 (a *cis*-Golgi marker), GRASP55 (a *medial*-Golgi marker) and Golgin97 (a TGN marker), respectively, along with TNKS1/2 antibody. We then used super-resolution Structured Illumination Microscopy (SIM) to study TNKS1 localization within the Golgi, as regular confocal microscopy appears to have insufficient resolution in our hand to determine *cis-/medial-/TGN* localization in the Golgi ribbon. The results indicated that endogenous Golgin45 appeared to have the highest co-localization index with TNKS (Pearson coefficients), followed by GM130 and GRASP55, while a TGN marker, Golgin97, showed the lowest co-localization with TNKS (Fig. 1f). Taken together, these results demonstrated that TNKS1 is likely to localize to the *cis*- and *medial*-Golgi compartments, and its targeting to the Golgi is dependent on Golgin45.

### TNKS2 plays a complementary role with TNKS1 in regulating Golgin45 protein stability during interphase

As TNKS1 protein stability was significantly enhanced upon Golgin45 depletion and inhibition of TNKS1 activity by XAV939 resulted in increased stability of Golgin45 protein^7^, TNKS1 depletion should lead to increased Golgin45 protein stability. Surprisingly, however, we found that knockdown of TNKS1 alone was not sufficient to stabilize Golgin45 to the level, which could be reached using XAV939 treatment (Fig. 1g; lane 2 and 5). Upon additional knockdown of TNKS1 and TNKS2, we were able to see similar increase of Golgin45 protein level, as shown in XAV939-treated cells (Fig. 1g; compare lane 4 and 5). As single knockdown of TNKS2 did not have any effect on Golgin45 protein stability, we conclude that TNKS2 may play a complementary role in the regulation of Golgin45 protein level. These results were further confirmed using confocal experiments, where we found that Golgin45 signal at the Golgi reached the highest level in cells depleted of both TNKS1 and TNKS2 (Fig. 1h).

### Inhibition of TNKS1 activity selectively increases the protein level of Golgin45, axin1, and TNKS1

**Fig. 2:**
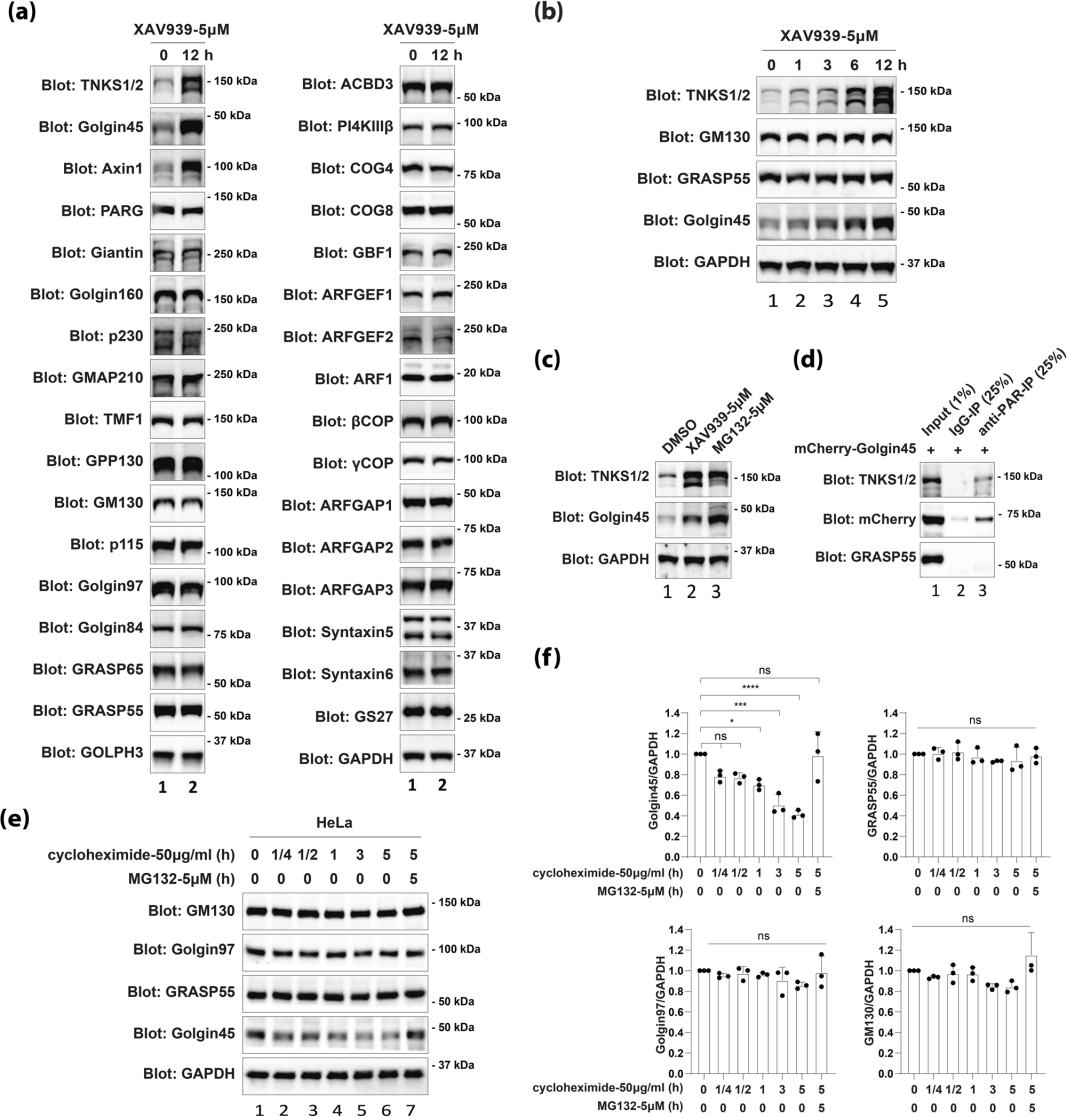
TNKS1 selectively regulates Golgin45 protein level by PARylation and subsequent proteasome-dependent degradation pathway. **a** TNKS1-dependent regulation is highly restricted to Golgin45 among Golgi-associated proteins. Western blots showing chemical inhibition of TNKS1 activity by XAV939 in HeLa cells for 12 h results in selective up-regulation of Golgin45 and TNKS1 protein level among the 30 Golgi-associated proteins tested here. Representative blots are shown and experiments were repeated two times. **b** Inhibition of TNKS1 activity results in a time-dependent increase of Golgin45 and TNKS1 protein level. HeLa cells were treated with XAV939 for the indicated times and lysed for analysis of endogenous proteins by SDS-PAGE and western blot analysis. Note that protein level of GM130, GRASP55, and GAPDH shows no significant change by XAV939 treatment. **c** Inhibition of proteasome and TNKS1 activity stabilizes Golgin45 and TNKS1 protein to a similar extent. HeLa cells were treated with either DMSO (control) or MG132 or XAV939 for 12 h, followed by lysis and western blotting with the indicated antibodies. Representative blots are shown and experiments were repeated three times. **d** anti-PAR IP from HeLa cells expressing mCherry-Golgin45 pulls down mCherry-Golgin45 and TNKS1/2 (positive control), but not GRASP55 (negative control). The protein extracts from HeLa cells expressing mCherry-Golgin45 were immunoprecipitated with anti-PAR or normal mouse IgG. These lysates and the immunoprecipitates were analyzed by western blotting using antibodies against the indicated proteins. Representative blots are shown and experiments were repeated three times. **e** Golgin45 protein half-life is very short, compared to other Golgi structural proteins. HeLa cells were treated with 50 μg/ml cycloheximide and MG132 for the indicated times. Endogenous GM130, Golgin97, GRASP55, Golgin45 and GAPDH were monitored by immunoblotting. **f** Quantification of endogenous GM130, Golgin97, GRASP55 and Golgin45 protein levels relative to GAPDH expression is shown. Statistical analysis was performed using one-way ANOVA with a Tukey’s post-hoc test (mean ± SD; n.s., not significant; *, *p* < 0.05; ***, *p* < 0.001; ****, *p* < 0.0001).

In order to confirm that TNKS1-regulated PARylation is specific on Golgin45 among Golgi-associated proteins^7^, we checked the protein levels of a large number of Golgi matrix proteins and SNAREs, etc, after inhibition of TNKS1 activity by XAV939. Using this screening, we aimed to establish that TNKS1 is unlikely to target other Golgi-associated proteins than Golgin45, as this is important to precisely determine the physiological role of TNKS1–Golgin45 interaction. To this end, HeLa cells were treated with 5 μM XAV939 or DMSO for 12 h and protein levels of various Golgi matrix proteins were examined by western blot analysis. The results (Fig. 2a) indicated that Golgi matrix proteins^14,15^, including GM130, p115, p230, Giantin, Golgin160, GMAP210, Golgin97, GRASP65/55, p24, COG complex and Golgi SNARE proteins (Syntaxin5, Syntaxin6, GS27) showed no significant change upon XAV939 treatment. However, Golgin45, axin1 and TNKS1 showed >5-fold increase in their protein level, suggesting that TNKS1-dependent regulation is likely to be restricted to Golgin45 at the Golgi complex, although TNKS1 is known to have as many as 30 different substrate proteins throughout the cell.

In addition, XAV939 treatment induced a linear increase in Golgin45 and TNKS1 protein level over the time-course of 12 h (Fig. 2b), and there was no significant difference between MG132, a proteasome inhibitor, and XAV939 treatment for their stabilizing effect on Golgin45 (Fig. 2c), confirming that TNKS1-mediated regulation of Golgin45 occurs at the protein level. In support of this observation, mCherry-Golgin45 and TNKS1/2 (positive control), but not GRASP55 (negative control) were detected in anti-PAR immunoprecipitate. (Fig. 2d).

As the results so far indicated that Golgin45 protein level is kept unusually low, due to TNKS1-dependent PARylation and subsequent degradation by the proteasome at steady state, we used a cycloheximide protocol to estimate protein half-life of Golgin45. The results showed that Golgin45 half-life is no longer than ~3 h, whereas other Golgin tethers tested, including GM130, GRASP55 and Golgin97, displayed less than ~10% reduction during the 5-hour chase period (Fig. 2e-f). These results further confirmed that Golgin45 has a very short protein half-life, compared to most other Golgi-associated proteins.

### TNKS1 contains four Arc domains that directly bind Golgin45 TBD, whose binding is partly regulated by a cell cycle-dependent kinase, Cdc2

**Fig. 3:**
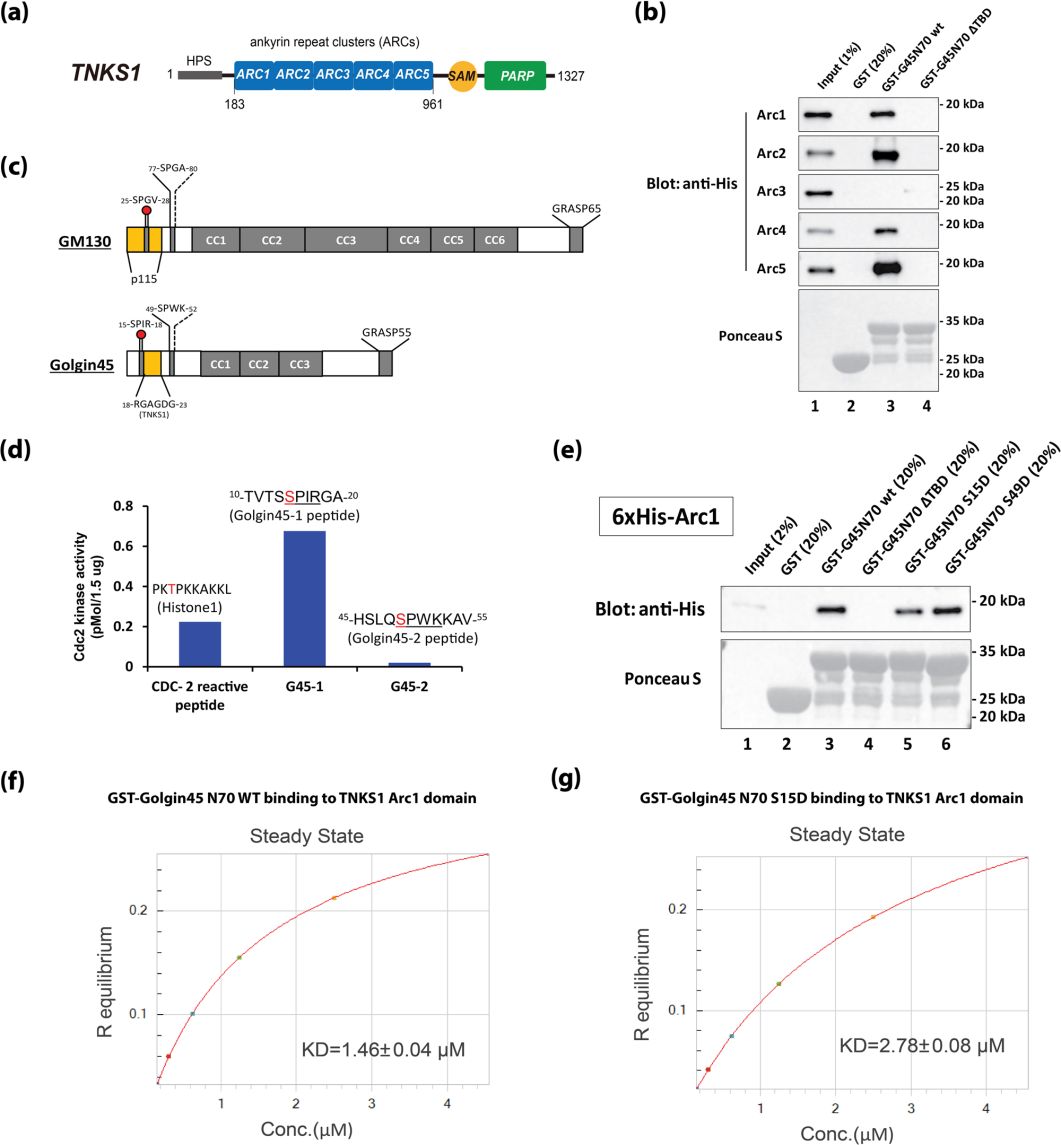
Golgin45 directly interacts with TNKS1, whose binding is partly regulated by a cell cycle-dependent kinase, Cdc2. **a** TNKS1 domain organization. TNKS1 contains amino-terminal HPS domain, five repeat clusters (ARCs), a SAM domain, and the PARP domain. **b** TNKS1 contains four domains (ArcI, II, IV, V) to bind Golgin45 directly. GST pull-down assays were performed using purified recombinant Arc domains of TNKS1 and GST-Golgin45 N-terminal domain (amino acid 1–70). **c** Schematic illustration of Golgin45 and GM130 domain organization and positions of the two putative consensus sequences for Cdc2 phosphorylation sites. **d** in vitro Cdc2 activity assay showing that S15 of ^15^SPIR18 consensus sequence-containing synthetic peptide gets phosphorylated by mitotic cytosol from HeLa cells. We used Histone-1 synthetic peptide (TPKK) as a positive control. **e** phospho-mimicking mutations reduces the binding interaction between Golgin45 and TNKS1. Purified His-tagged Arc1 was incubated with GST-Golgin45 N70 wt or ΔTBD or S15D or S49D, respectively, and bound fractions were analyzed by western blots. **f**, **g** Steady-state analysis of the interaction between Golgin45 N70 WT and TNKS1 Arc1 domain, using Biolayer interferometry assays. The experiments were repeated three times.

TNKS1 is known to contain five ARC (Ankyrin Repeat Cluster) domains in its N-terminal region, followed by SAM (Sterile α-Motif) domain for oligomerization and PARP (Poly(ADP-ribose) Polymerase) domain for enzymatic activity (Fig. 3a)^16^. In order to more accurately characterize Golgin45 interaction with TNKS1, we expressed and purified recombinant ARC domains of TNKS1 from bacteria. These individual ARC domains of human TNKS1 were then incubated with Golgin45 N-terminal 70 amino acids fused to GST, in order to study the binding specificity. The results showed that four Arc domains (Arc I, II, IV, V) bound well to Golgin45 N-terminal domain fused to GST, but not to the Golgin45 N-terminus ΔTBD (Fig. 3b), indicating that TNKS1 contains at least four binding sites for Golgin45 Ankyrin repeat motifs.

We have previously shown that Golgin45 and GM130 play a partly redundant role in Golgi structure maintenance^12^. Although these two proteins appear to be unrelated phylogenetically, they share similar arrangement for binding partner interaction, such as GRASPs and Syntaxin5^8,10^. As N-terminal domain of GM130 mediates its binding to another vesicle-binding Golgin tether, p115, in a cell cycle-dependent manner via Cdc2^17^, we reasoned that Golgin45-TNKS1 interaction could be also subjected to Cdc2-dependent regulation. Upon close examination of neighboring sequences in Tankyrase Binding Domain (TBD) of Golgin45, we found two Cdc2-consensus sequences, ^15^SPIR^18^ and ^49^SPWK^52^, in Golgin45 N-terminus (Fig. 3c)^17^. Using Cdc2 activity assay and synthetic peptides, we found that ^15^SPIR^18^ sequence (and control Histone-1 peptide) showed significantly higher Cdc2 activities, compared to ^49^SPWK^52^ sequence (Fig. 3d; Supplementary Fig. 1c).

We then mutated the Serine residues to Aspartic acid in ^15^SPIR^18^ and ^49^SPWK^52^ 18 and tested whether the phospho-mimicking mutations could negatively influence the binding between Golgin45 and TNKS1. Purified His-tagged ARC1 was incubated with GST-Golgin45 N70 wt or ΔTBD or S15D or S49D, respectively, and bound fractions were analyzed by western blots. The results showed that S15D mutated GST-Golgin45 pulled down significantly reduced amount of purified ARC1 domain, compared to WT Golgin45 (Fig. 3e; compare lane 3 and 5). Consistent with Cdc2 activity assays, GST-Golgin45 S49D mutant didn’t show any difference to the WT, and GST-Golgin45 ΔTBD failed to bind purified ARC1 (Fig. 3e; lane 6 and 4, respectively).

We then used Biolayer interferometry (BLI) assays to determine their precise binding constants to further verify the effect of S15D mutation. The results showed that the dissociation constant (Kd) between control Golgin45 and TNKS1 Arc1 domain was approximately 1.46 ± 0.04 μM (*R*^2^ = 1), whereas it was reduced to 2.78 ± 0.08 μM (*R*^2^ = 1) for Golgin45 S15D mutant (Fig. 3f, g). Taken together, these results suggest that TNKS1–Golgin45 interaction is subjected to Cdc2-dependent regulation in a cell cycle-dependent manner, like GM130-p115 interaction, although their binding interaction is not abrogated to the extent, as in GM130-p115 interaction.

### Golgin45 knockdown slows down secretion of both small and large cargoes, while increased Golgin45 affects small cargo only

To study the physiological role of TNKS1-dependent regulation of Golgin45, we then asked whether secretory function of the Golgi remains normal in XAV939-treated cells. To this end, a FM4 (four repeats of a FKBP-self aggregating mutant) domain-dependent conditional aggregation system was used for controlled-release of cargo-wave along the secretory pathway^19^, in order to study whether anterograde protein secretion was affected in these cells. We used a modified version of the transferrin receptor (TfR-FM4-SNAP) containing FM domain repeats that spontaneously aggregates in the ER to prevent its exit. Exit can then be triggered by FM ligand AP21998 (also known as D/D solubilizer) drug-induced depolymerization^19,20^. Briefly, HeLa cells were transfected with a plasmid encoding Transferrin receptor fused to FM4, followed by SNAP, TfR-FM4-SNAP^21^ and XAV939 (5 μM) treatment for 18 h. The cargoes were then released by adding D/D solubilizer that is known to solubilize and release TfR-FM4-SNAP cargo from the ER for the indicated times^20^, and surface expression of TfR was estimated by surface biotinylation protocol using sulfo-NHS-LC-biotin, as described previously^12^.

**Fig. 4:**
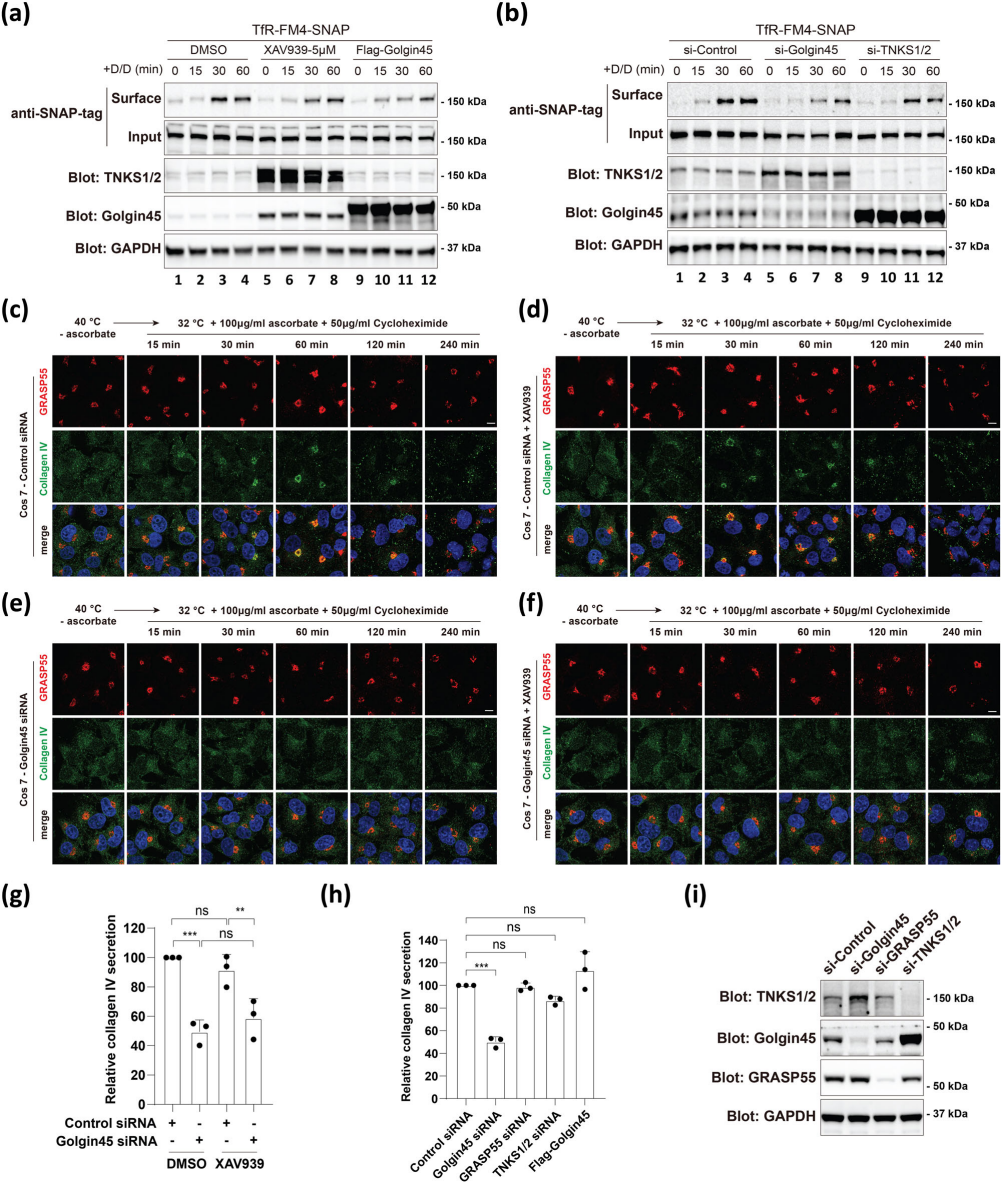
TNKS1-dependent regulation of Golgin45 moderately influences both small and large oversize cargo secretion, but does not completely inhibit protein secretion. **a** TNKS1 inhibition by XAV939 treatment or overexpression of Flag-Golgin45 moderately reduces anterograde small cargo trafficking. HeLa cells were transfected with Transferrin receptor fused to FM4 (conditional aggregation domain) and SNAP (TfR-FM4-SNAP), followed by XAV939 treatment overnight, or co-transfected with TfR-FM4-SNAP and Flag-Golgin45 overnight. Cells were then treated with cycloheximide for 2 h, prior to induction of synchronized protein secretion by treatment with D/D solubilizer drug for the indicated times. At the indicated time points, the cells were placed on ice and subjected to surface biotinylation using sulfo-NHS-LC-biotin for 30 min. The cells were then lysed, subjected to pulldown with streptavidin-agarose and analyzed by western blot. Representative blots are shown and experiments were repeated three times. **b** Golgin45 or TNKS1/2 depletion also moderately reduces anterograde small cargo trafficking. After 48 h transfection with indicated siRNAs, HeLa cells were transfected with TfR-FM4-SNAP overnight. Cells were then treated with cycloheximide for 2 h, prior to induction of synchronized protein secretion by treatment with D/D solubilizer drug for the indicated times. The amount of TfR-FM4-SNAP on the cell surface was estimated by surface biotinylation protocol. Representative blots are shown and experiments were repeated three times. **c**–**f** Golgin45 depletion significantly inhibits Collagen IV transport, while TNKS1 inhibition by XAV939 does not influence Collagen IV transport. Immunofluorescence staining of Collagen IV and a Golgi marker (GRASP55) was performed in WT or Golgin45 knockdown COS7 cells treated with or without XAV939 under folding block (40 °C, 3 h) without ascorbate and transport pulse condition, which was later induced by shifting cells to 32 °C in the presence of 100 mg/mL ascorbate and 50 µg/ml Cycloheximide for the indicated times. Scale bar, 10 µm. **g** Collagen IV secretion quantified by ELISA under various conditions. Collagen IV secretion is inhibited by Golgin45 depletion, but not affected by inhibition of TNKS1 activity. WT or Golgin45 knockdown COS7 cells were treated with or without XAV939. Cell supernatants were collected after 6 h of traffic pulse of Collagen IV and used for Collagen IV ELISA. Statistical analysis was performed using two-way ANOVA with a Tukey’s post-hoc test for multiple comparisons (mean ± SD; n.s., not significant; **, *p* < 0.01; ***, *p* < 0.001). **h** Collagen IV secretion is not affected by knockdown of GRASP55, double knockdown of TNKS1/2 or Golgin45 overexpression. COS7 cells were transfected with the indicated siRNAs for 72 h or transfected with Flag-Golgin45 overnight. Cell supernatants were collected after 6 h of traffic pulse of Collagen IV and used for Collagen IV ELISA. Statistical analysis was performed using one-way ANOVA with a Tukey’s post-hoc test (mean ± SD; n.s., not significant; ***, *p* < 0.001). **i** Immunoblot analysis showing knockdown efficiency of indicated siRNA used in Collagen IV secretion experiments.

The results indicated that TfR-FM4-SNAP secretion was moderately reduced in XAV939-treated HeLa cells, compared to the control cells (Fig. 4a; compare lane 1–4 and lane 5–8), suggesting that increased Golgin45 protein level may be moderately detrimental to TfR-FM4-SNAP trafficking through the Golgi. This observation was confirmed in cells overexpressing FLAG-tagged Golgin45 (Fig. 4a; compare lane 1–4 and lane 9–12), which showed even more reduction in secretion, further supporting the observation that increased Golgin45 protein level may moderately influence small cargo trafficking through the Golgi. Interestingly, knockdown of Golgin45 or TNKS1/2 (which increases Golgin45 protein stability) also appeared to reduce TfR-FM4-SNAP trafficking through the Golgi (Fig. 4b). During three repeated experiments, we observed a similarly moderate, but highly consistent reduction in TfR-FM4-SNAP secretion to the cell surface.

To more thoroughly examine the secretory function of the Golgi in response to various Golgin45 protein level, we studied secretion of a large cargo, collagen IV, in COS 7 cells treated with DMSO vs. XAV939 for 12 h. At 40 °C (and in the absence of ascorbate), collagen proteins cannot fold completely so they are trapped in the ER. Collagen synthesis is regulated by ascorbate. When cells were shifted to 32 °C with the addition of ascorbate, collagen proteins fold and leave the ER. To this end, WT or Golgin45 knockdown COS7 cells treated with DMSO vs. XAV939 for 9 h were subjected to folding block (40 °C, 3 h) without ascorbate. Collagen secretion was induced by combination of temperature shift (40 °C → 32 °C) and ascorbate treatment in the presence of cycloheximide, as described previously^22,23^. Secretion of collagen IV was studied by staining with antibodies against endogenous collagen IV and anti-GRASP55 antibody as a Golgi marker and confocal microscopy as well as a commercial ELISA kit for collagen IV secretion.

The results showed that secretion of collagen IV showed no significant influence in DMSO vs XAV939-treated COS7 cells (Fig. 4c, d for confocal; Fig. 4g for ELISA), suggesting that large cargo secretion is not affected by inhibition of TNKS1 activity.

We have previously shown that Golgin45 knockdown significantly disrupt the Golgi ribbon and results in a reduced number of cisternae per Golgi stack^10^. Consistent with this earlier study, Golgin45 depletion resulted in ~50% reduction in collagen IV secretion (Fig. 4e for confocal; Fig. 4g for ELISA), whereas XAV939 treatment in Golgin45-depleted cells didn’t cause a significant change in Collagen IV secretion, compared to DMSO-treated Golgin45 KD cells (Fig. 4f for confocal; Fig. 4g for ELISA).

In addition, knockdown of GRASP55, double knockdown of TNKS1/2 or Golgin45 overexpression didn’t have a significant influence on collagen IV secretion (Fig. 4h, i). Taken together, these results suggest that (i) increased Golgin45 protein in XAV939-treated cells or cells overexpressing Golgin45 reduces small cargo secretion through the Golgi; (ii) Golgin45 knockdown moderately influences both small and large cargo protein secretion through the Golgi.

### Protein glycosylation is significantly influenced upon inhibition of TNKS1 activity

Since TNKS1-mediated regulation of Golgin45 protein level appears to only moderately influence secretory function of the Golgi, we next asked whether TNKS1 inhibition may regulate other Golgi function, such as protein glycosylation. Previous work by Wang and colleagues had shown that double depletion of GRASP65 and GRASP55 results in significant alteration of protein glycosylation^24^, based on glycomic analysis using mass spectrometry.

In order to quantitatively analyze the effect of TNKS1-mediated regulation of Golgin45 on protein glycosylation at the Golgi, we used mass spectrometry to measure the global efficiency of glycoprotein maturation in XAV939-treated cells. In particular, the efficiency of mannose trimming for high mannose-type N-glycans and in the formation of sialylated complex-type N-glycans were closely monitored and analyzed^25^.

To this end, we prepared N-glycanase-treated tryptic peptides from equal amounts of DMSO (control) or XAV939-treated HeLa cell protein extracts, as described in the methods. In order to measure the relative changes in different glycan species, we used permethylated maltotetraose as an internal standard by spiking 5 pmol of isotopically labeled maltotetraose to each sample prepared from DMSO or XAV939-treated cells. For normalization of mass spec data, the area of each isotope peak was integrated and the area of glycans for quantification was calculated as the sum of peak areas obtained from the isotope envelope. Then, glycan areas were normalized by dividing them by the peak area of the internal standard spiked to the sample.

**Fig. 5:**
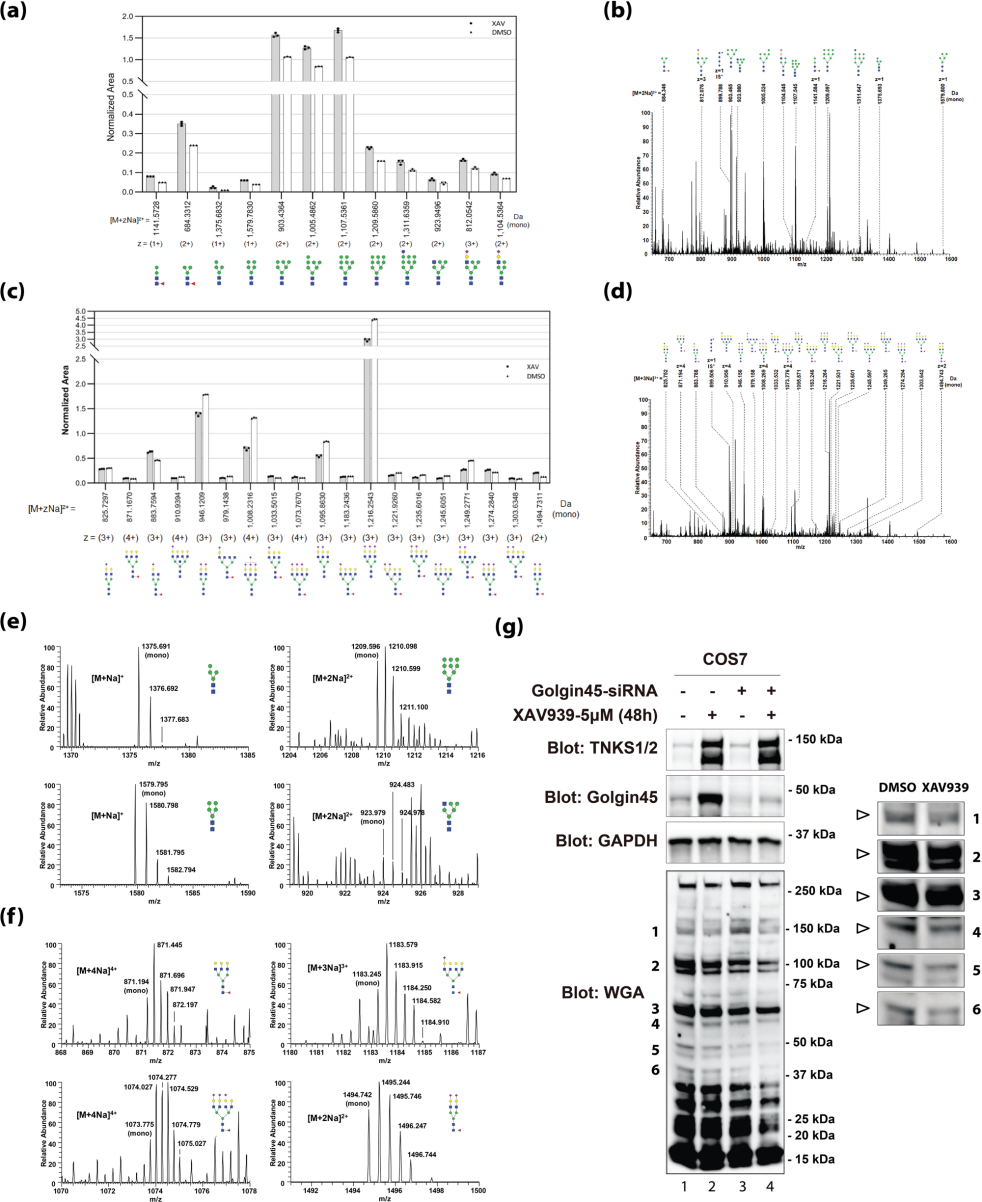
Glycomic analysis reveals a significant alteration of protein glycosylation in XAV939-treated HeLa cells. **a** Comparison histogram between normalized XAV peak area and normalized DMSO peak area for high mannose and hybrid N-linked glycans. z: charge state. **b** Full mass spectrum of N-linked glycans from XAV. An asterisk denotes an isotopic sign. IS: internal standard. **c** Comparison histogram between normalized XAV peak area and normalized DMSO peak area for complex N-linked glycans. Low-abundance glycans, eight non-sialylated complex N-linked glycans with a normalized area of less than 0.09, were excluded from the histogram. **d** Full mass spectrum of N-linked glycans from DMSO. **e** High-resolution mass spectra of 4 representative low-abundance high mannose N-linked glycans from XAV939 sample. **f** High-resolution mass spectra of 4 representative low-abundance complex-type N-linked glycans from DMSO sample. **g** WGA lectin blots indicate altered N-glycosylation of glycoproteins upon inhibition of TNKS1 activity by XAV939 treatment or Golgin45 knockdown or combination of these two treatments in COS7 cells. Cells were treated with either DMSO or XAV939 alone for 48 h (lane 1 and 2) or treated with either DMSO or XAV939 after Golgin45 knockdown (lane 3 and 4) were lysed in SDS-sample buffer and subjected to SDS-PAGE and lectin blotting using HRP-conjugated WGA (see magnified insets for close-up view of the WGA bands; empty triangle for decreased signal). The protein level of TNKS1/2 and Golgin45 after the indicated treatments are also shown.

Strikingly, the results showed that high mannose-type N-glycans of various species significantly increased in XAV939-treated sample over the wild-type control sample (Fig. 5a, b**;** Supplementary Fig. 2a; also see Fig. 5e for representative full spectra of low-abundance high mannose-type glycan species), whereas sialylated complex-type N-glycans were notably decreased in XAV939-treated sample (Fig. 5c, d**;** Supplementary Fig. 2b; also see Fig. 5f for representative full spectra of low-abundance complex-type glycan species).

These glycomics results were further verified using WGA lectin as probes to detect changes in global N-glycosylation^26^. HeLa cells treated with either DMSO or XAV939 for 48 h were lysed in SDS-sample buffer and subjected to SDS-PAGE and WGA lectin blot analysis. Consistent with the glycomic data, the results showed that XAV939 treatment significantly altered WGA binding profiles, compared to DMSO-treated control samples (Fig. 5g**;** compare lane 1 and 2; empty triangle for decreased signal in magnified insets to the right), further supporting the glycomics results that XAV939 treatment influences protein glycosylation.

In order to determine how much of these altered protein glycosylation may be attributed to regulation of Golgin45 protein stability by TNKS1, we also studied whether XAV939 treatment in Golgin45 knockdown cells fails to induce a similar change in protein glycosylation, compared to XAV939 treatment alone. However, the results provided a complex phenotype that was elusive in supporting the direct involvement of Golgin45 protein stability via TNKS1 for regulation of protein glycosylation. In particular, XAV939 treatment in Golgin45-depleted cells further decreased overall WGA binding in the lectin blots over Golgin45 knockdown alone (Fig. 5g**;** compare lane 3 and 4).

### FRAP experiments indicate that TNKS1 activity affects dynamics of glycosyltransferase trafficking at the Golgi

We then posited that dynamics of glycosyltransferease trafficking at the Golgi might have been altered in these cells. Golgi glycosyltransferases are known to undergo constant recycling between the ER and the Golgi as well as among the Golgi stacks^27,28^. To this end, fluorescence recovery after photobleaching (FRAP) technique was used to study whether TNKS1 inhibition by XAV939 or Golgin45 overexpression may influence dynamics of glycosyltransferase trafficking at the Golgi.

**Fig. 6:**
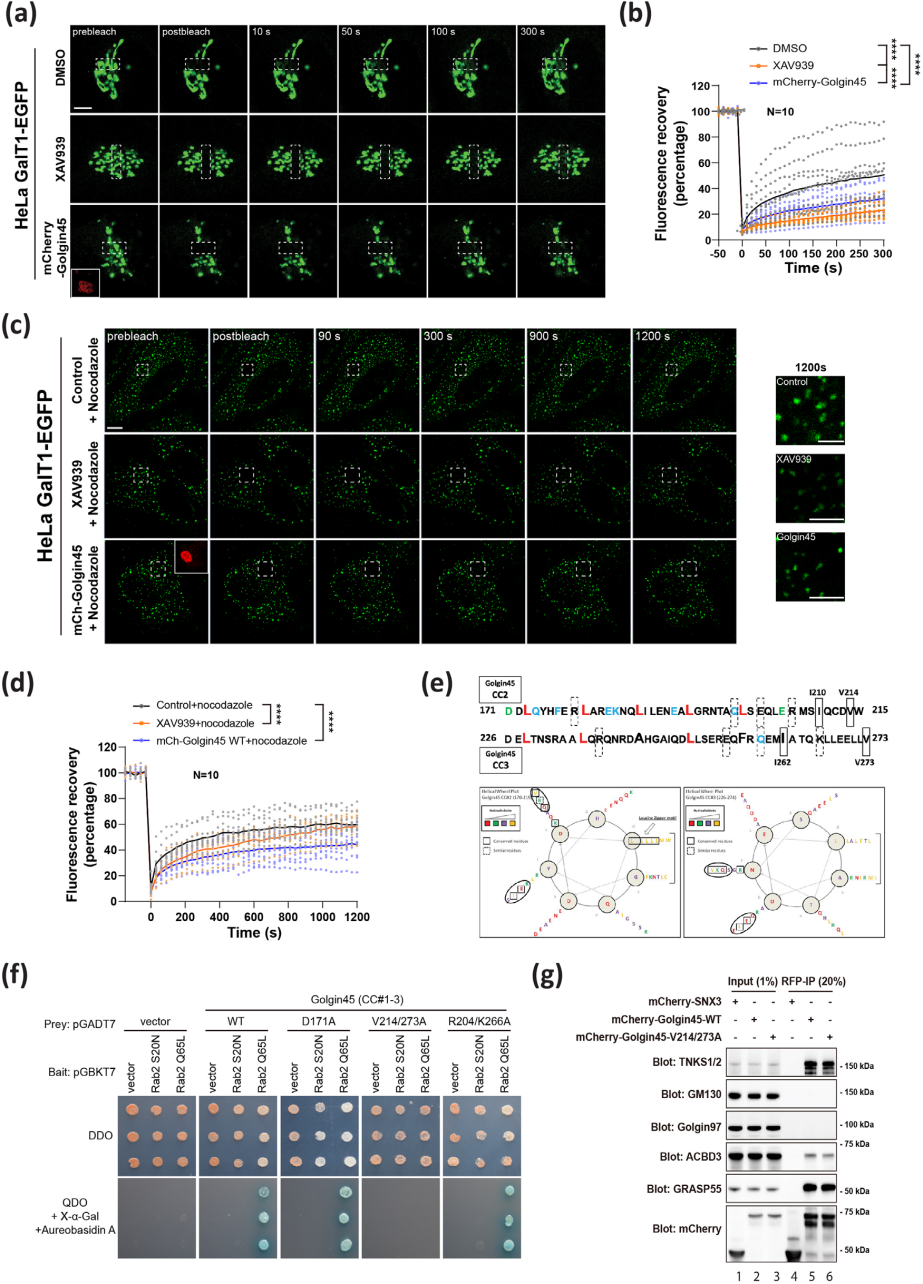
Inhibition of TNKS1 activity or Golgin45 overexpression greatly influences dynamics of GalT1-EGFP trafficking to the Golgi, as shown by FRAP experiments. **a** HeLa cells expressing GalT1-EGFP were treated with either DMSO (control) or 5 μM XAV939 or a plasmid encoding mCherry-Golgin45 for 12 h. A rectangular area of the Golgi was bleached as indicated. Recovery of fluorescence was observed by live cell imaging, as described in the methods. Representative images of the indicated times are shown. Scale bar, 10 μm. **b** The ratio of fluorescence of the bleached area to an adjacent unbleached area was measured for each time point, normalized to the initial values and plotted, as described in the methods. Statistical analysis was performed using two-tailed, paired *t* test (*N* = 10, ****, *p* < 0.0001). **c** HeLa cells expressing GalT1-EGFP were treated with either DMSO (control) or 5 μM XAV939 or a plasmid encoding mCherry-Golgin45 for 12 h. Next day, nocodazole was added to the medium of all samples for 3 h to induce complete fragmentation of the Golgi ribbon. A square area of the Golgi was bleached as indicated. Recovery of fluorescence was observed by live cell imaging. Representative images of the indicated times are shown. Scale bar, 10 μm. Scale bars for magnified insets are 5 μm. **d** The ratio of fluorescence of the bleached area to an adjacent unbleached area was measured for each time point, normalized to the initial values and plotted, as described in the methods. Statistical analysis was performed using two-tailed, paired *t* test (*N* = 10, ****, *p* < 0.0001). **e** Helical wheel plots predict CC2 and CC3 of Golgin45 share a striking similarity (circled residues). **f** Yeast two-hybrid assays strongly suggest that V214/273A mutation completely abrogate the interaction between Golgin45 CC domains and Rab2-GTP. Growth of yeast cells expressing Rab2-Q65L (GTP-locked mutant) or Rab2-S20N (GDP-locked mutant) bait or a pGBKT7 bait vector with the following preys: Golgin45 (CC#1-3) WT or mutants (D171A, V214/273A or R204/K266A) or pGADT7 (prey vector as a negative control) were challenged on agar plates depleted of tryptophan and leucine (DDO plates, upper panel) or depleted of tryptophan, leucine, histidine and adenine, plus 40 μg/ml X-α-Gal and 0.2 μg/ml Aureobasidin A (QDO/ X-α-Gal/AbA plates, bottom panel) by spotting three independent transformants on different plates. **g** Immunoprecipitation experiments showing that V214/273 A mutation does not inhibit Golgin45 interaction with various binding partners, including GRASP55, TNKS1 and ACBD3. The protein extracts from HeLa cells transfected with mCherry tagged Sorting Nexin 3 (mCherry-SNX3 as a control), mCherry-Golgin45 or mCherry-Golgin45-V214/273 A were immunoprecipitated with anti-RFP agarose beads. These lysates and the immunoprecipitates (anti-RFP IPs) were analyzed by western blotting using anti-mCherry antibody and antibodies against the indicated proteins.

Initially, we studied whether XAV939 treatment or Golgin45 overexpression may cause moderate fragmentation of the Golgi ribbon and how this may potentially influence protein glycosylation (Fig. 4). Thus, HeLa cells were transiently transfected with β(1, 4)-galactosyltransferase fused to GFP (GalT1-GFP) and treated with either DMSO (control) or 5 μM XAV939 or transfected with a plasmid encoding mCherry-Golgin45 for 12 h. We then performed FRAP experiments by photobleaching a rectangular area of the Golgi ribbon, as shown in Fig. 6a.

The results of FRAP experiments revealed that both XAV939 and Golgin45 overexpression cause a significant delay in the recovery of fluorescent signals in the bleached Golgi area, compared to the control (Fig. 6a, b), suggesting that, like many other Golgin tethers, increased expression of Golgin45 results in moderate fragmentation of the Golgi ribbon structure. As ribbon fragmentation by nocodazole treatment has been shown to have a negligible effect on small cargo secretion^29^ and both TfR-FM4-SNAP and collagen IV secretion was not affected in XAV939-treated cells (Fig. 4a–d), we conclude that moderate fragmentation of the Golgi ribbon by increased Golgin45 protein level may not significantly influence cargo trafficking and protein glycosylation.

In order to investigate the effect of Golgin45 protein stability in glycosyltransferase trafficking among the Golgi stacks, we then used nocodazole-treated cells for all subsequent FRAP experiments to more accurately characterize any subtle change in the trafficking dynamics of Golgi glycosyltransferases. Thus, HeLa cells were transfected with GalT1-GFP alone, followed by XAV939 treatment or co-transfected with mCherry-Golgin45 overnight. Next day, nocodazole was added to the medium of all samples for 3 h to induce complete fragmentation of the Golgi ribbon. During FRAP experiments, a few Golgi stacks were photobleached and fluorescent recovery in the photobleached stacks was recorded and analyzed by time-lapse microscopy, as shown in Fig. 6c.

Strikingly, we found that XAV939 treatment caused a significant delay in the fluorescent recovery of GalT1-GFP, and Golgin45 overexpression alone similarly resulted in even further delay in the fluorescent recovery, as shown in Fig. 6c, d. Overall, these results suggested that TNKS1-dependent regulation of Golgin45 protein stability is likely to influence trafficking dynamics of Golgi glycosyltransferases.

### Golgin45-Rab2-GTP interaction plays a crucial role in Golgin45-dependent regulation of GalT1-GFP trafficking dynamics

Although our results so far demonstrated that TNKS1-mediated regulation of Golgin45 protein stability significantly affects glycosyltransferase trafficking and subsequent protein glycosylation at the Golgi, there were two limitations in understanding the exact mechanism of this novel regulation. First, inhibition of TNKS1 activity by XAV939 treatment likely influences a number of other TNKS1 substrates, which include as many as thirty different proteins^30^, making interpretation of the FRAP and glycomic results complicated and potentially misleading. Secondly, the mechanism by which Golgin45 protein level may influence trafficking dynamics of glycosyltraferases at the Golgi was still elusive.

To circumvent this challenging issue and better understand the mechanistic underpinning of these findings, we then hypothesized that the change in GalT1-GFP trafficking dynamics upon increased Golgin45 expression may be mediated by Golgin45-Rab2-GTP interaction. As Golgin-Rab-GTPase interaction has long been known to regulate vesicular transport around the Golgi^14,31^, we decided to look for Golgin45 mutants that specifically abrogate its interaction with Rab2-GTP, in order to better demonstrate that altered glycosyltransferase trafficking via TNKS1-mediated regulation of Golgin45 protein level may involve specific interaction between Golgin45 and Rab2-GTP.

First, we confirmed that purified recombinant Golgin45 coiled-coil (CC) domains selectively interact with Rab-2-GTP by pull-down assays^8^, as shown in Supplementary Fig. 3a. During amino acid sequence analysis of Golgin45 CC domains, we found that CC2 and CC3 of Golgin45 share a striking similarity, when examined using helical wheel plots (Fig. 6e). In particular, we found that ‘VRQ’ sequence of CC2 and ‘VKQ’ sequence of CC3 (circled residues) aligned well along the z-axis of the CC domains, suggesting that these sequences may be conserved for a certain unknown function or binding interaction.

Furthermore, upon deletion of CC3, GST-Rab2-GTPγS showed reduced binding to the 6xHis tagged recombinant Golgin45 CC(1-2) domains (Supplementary Fig. 3b), yet there was still relatively strong interaction between the two recombinant proteins, providing an evidence that Golgin45 CC2 may harbor an additional binding site for Rab2-GTP.

Based on these clues, we mutated the conserved two valine residues to alanine (V214/273A) and the two positively charged residues to alanine (R204A/K266A) to test whether these mutations can selectively inhibit Golgin45-Rab2-GTP interaction. As a control, we also included D171A mutant, which had been shown to selectively abrogate Golgin45 interaction with Syntaxin5^10^. We then used yeast two-hybrid assays to test Golgin45 CC mutants for its interaction with Rab2-GTP.

As shown in Fig. 6f, the control Golgin45 CC domain selectively interacted with Rab2-GTP, whereas V214/273A mutation completely blocked the interaction between Golgin45 CC domains and Rab2-GTP. In addition, neither D171A nor R204A/K266A mutation showed any effect, suggesting that V214/273A mutation selectively inhibit Golgin45 interaction with Rab2-GTP. This finding was further confirmed using purified recombinant proteins and GST pull-down assays, as shown in Supplementary Fig. 3c.

In order to exclude the possibility that V214/273A mutation may interfere with Golgin45 interaction with its various interacting partners, including TNKS1, GRASP55 and ACBD3^8,9^, we performed immunoprecipitation experiments. To this end, HeLa cells were transfected with either mCherry-SNX3 (a negative control) or mCherry-Golgin45-WT or -V214/273A mutant overnight. Cells were then lysed and subjected to immunoprecipitation using anti-RFP antibody. The results showed that V214/273A mutation did not have any influence on Golgin45 interaction with TNKS1, GRASP55 and ACBD3, compared to mCherry-Golgin45-WT (Fig. 6g), respectively. As expected, Golgin45 did not pull down GM130 or Golgin97, while SNX3 didn’t pull down any of the Golgi proteins tested in the experiments. Prior to the FRAP experiments, we also confirmed that V214/273 A mutation doesn’t influence Golgi localization of mCherry-Golgin45 (Supplementary Fig. 3d).

We then performed FRAP assays using either mCherry-Golgin45 WT or mCherry-Golgin45 V214/273A mutant in knockdown-rescue experiments. Briefly, HeLa cells were transfected with Golgin45 RNAi oligos for 48 h to deplete Golgin45 protein. These Golgin45 knockdown cells were then transfected with GalT1-GFP alone or co-transfected with mCherry-Golgin45 WT or mCherry-Golgin45 V214/273A mutant overnight. Cells were then treated with nocodazole for 3 h to fragment the Golgi ribbon, followed by the standard FRAP protocol to investigate whether Golgin45-Rab2-GTP interaction may play a role for Golgin45-dependent regulation of GalT1-GFP trafficking.

**Fig. 7:**
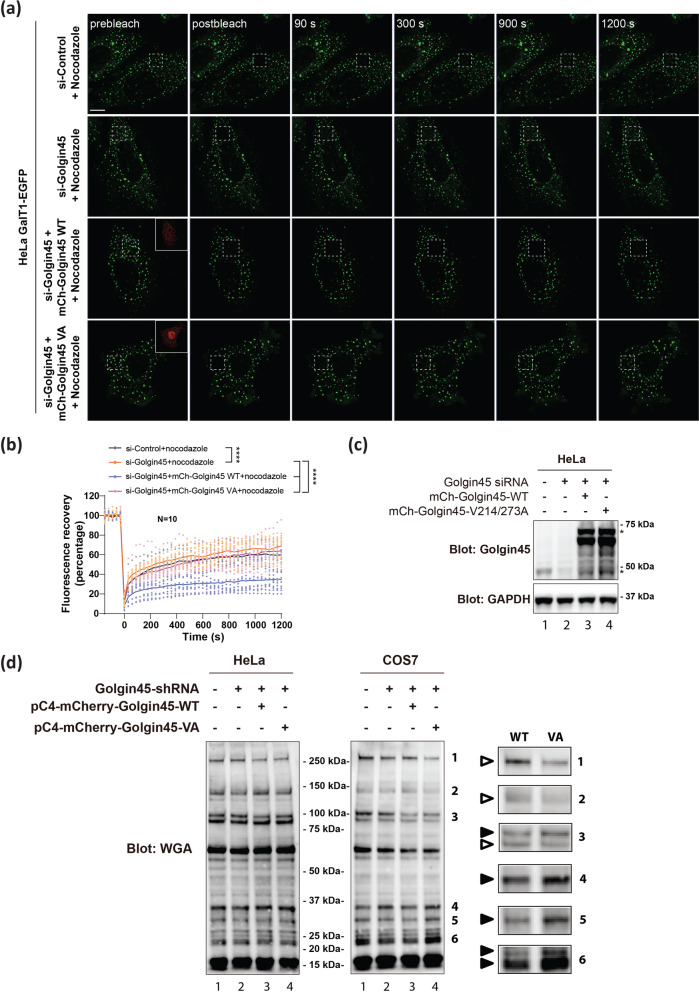
Golgin45 protein level dictates GalT1-EGFP trafficking to the Golgi in Rab2-GTP-dependent manner. **a** HeLa cells were transfected with Golgin45 RNAi oligos for 48 h to deplete Golgin45 protein. Golgin45 knockdown cells were then co-transfected with GalT1-GFP plus mCherry-Golgin45 wt or mCherry-Golgin45 V214/273A mutant overnight. Cells were then treated with nocodazole for 3 h to fragment the Golgi ribbon, followed by the standard FRAP protocol. A square area of the Golgi was bleached as indicated. Recovery of fluorescence was observed by live-cell imaging. Representative images of the indicated times are shown. Scale bar, 10 μm. **b** The ratio of fluorescence of the bleached area to an adjacent unbleached area was measured for each time point, normalized to the initial values and plotted, as described in the methods. Statistical analysis was performed using two-tailed, paired *t* test (*N* = 10, ****, *p* < 0.0001). **c** Immunoblot analysis of knockdown and rescue efficiency of Golgin45 used in FRAP experiments. **d** WGA lectin blots showing the striking effect of Golgin45 WT vs. Rab2-binding deficient Golgin45 mutant overexpression in Golgin45 stable knockdown cells on protein glycosylation. HeLa and COS7 cells were treated with Golgin45 shRNA by lentivirus transduction to establish a stable Golgin45 knockdown cell lines. These stable cells were transfected with either mCherry-Golgin45 WT or mCherry-Golgin45 VA mutant plasmid for 24 h, followed by lysis in SDS-sample buffer and WGA lectin blots. Note that COS7 cells showed more prominent difference between WT vs. VA in WGA blots, which is denoted by magnified insets, shown to the right (empty triangle=reduced signal; filled triangle=increased signal).

The results showed that knockdown of Golgin45 moderately increased the fluorescent recovery, compared to the control samples during the FRAP experiments (Fig. 7a, b; compare black and orange data points). Upon the rescue transfection with the control mCherry-Golgin45, there was a drastic decrease in the fluorescent recovery (Fig. 7a-b; compare orange and blue data points). Because the rescue transfection actually resulted in overexpression of mCherry-Golgin45 (Fig. 7c), it made sense that the rescued cells showed a similar decrease of fluorescent recovery, comparable to the cells simply overexpressing mCherry-Golgin45 (Fig. 6c, d).

Importantly, there was a striking difference between the control Golgin45 and Golgin45 V214/273A mutant in their ability to regulate GalT1-GFP trafficking in the knockdown background. The rescue transfection with the control mCherry-Golgin45 in knockdown background severely interfered with fluorescent recovery, whereas this delay was significantly less prominent in cells transfected with Golgin45 V214/273A mutant (Fig. 7a-b; compare purple and blue data points), suggesting that Golgin45-Rab2-GTP interaction may play a pivotal role in Golgin45-mediated modulation of GalT1-GFP trafficking at the Golgi.

In support of these findings, WGA lectin blots of similar knockdown and rescue experiments using mCherry-Golgin45 WT and mCherry-Golgin45 V214/273A mutant showed significantly different protein glycosylation pattern between cells expressing Golgin45 WT and Golgin45 VA (Fig. 7d**;** Supplementary Fig. 3e) in both HeLa and COS7 cells (see magnified insets of several bands in Fig. 7d for WGA blots from COS7 experiments), although this was much more obvious in COS7 cells. Taken together, these results unequivocally showed that Golgin45-Rab2-GTP interaction is required for TNKS-mediated regulation of glycosyltransferease trafficking and protein glycosylation via modulation of Golgin45 protein stability.

## Discussion

Our study reveals a previously unrecognized connection between TNKS1, a regulator of Wnt/β-catenin signaling pathway, and protein glycosylation at the Golgi complex. Since glycosylation is known to play important roles during development and oncogenic transformation^32,33^, the connection between protein glycosylation and Wnt/β-catenin signaling is not surprising. Studies have already shown that Wnt/β-catenin signaling directly targets and regulates *DPAGT1* gene, the dolichol-P-dependent *N-*acetylglucosamine-1-phosphate-transferase (GPT), which initiates the synthesis of the lipid-linked oligosaccharide precursor for protein *N-*glycosylation in the endoplasmic reticulum^34–36^.

Whereas Wnt/β-catenin-dependent regulation of *DPAGT1* gene occurs at the transcriptional level for glycosylation of nascent polypeptides in the endoplasmic reticulum, TNKS1-mediated post-translational regulation of Golgin45 seemingly controls glycosyltransferase trafficking and subsequent protein glycosylation at the Golgi complex.

Glycosylations, such as increased sialylation and multi-branched N-glycans, have been shown to play important roles during development^32,33^, and aberrant N-glycosylations have long been regarded as a hallmark of oncogenic transformation and shown to contribute to increased invasiveness and metastatic potential^37–41^. Therefore, it makes sense that a regulator of Wnt/β-catenin signaling also modulates protein glycosylation at the Golgi, albeit in an unexpected way.

Given the large number of TNKS1 substrates, all of which would be influenced by XAV939 treatment, it is clear that XAV939 may influence protein glycosylation in more complex manner, which creates difficulty in understanding how regulation of Golgin45 protein stability by TNKS1 may influence glycosyltransferase trafficking and protein glycosylation. Nonetheless, our results using Rab2-binding deficient Golgin45 mutant allowed much more straightforward interpretation of the glycomic and FRAP data in our study.

On the other hand, exactly how does Golgin45-Rab2 interaction affect trafficking of glycosyltransferases at the Golgi, which is known to be mostly mediated by COPI vesicles^14,42,43^? While it may not be unexpected that protein level of a Rab-binding Golgin tether can influence glycosyltransferase trafficking, it was rather counter-intuitive in several ways, as the conventional model in the field would suggest that increased binding of Golgin45-Rab2-GTP would naturally lead to enhanced vesicle tethering and fusion of glycosyltransferase containing transport carriers to the Golgi^14,44^.

Golgi glycosyltransferases have been known to undergo constant recycling between the ER and the Golgi^27,28^. Rab2-GTP had previously been shown to play an essential role in the maturation of pre-Golgi intermediates and anterograde transport between pre-Golgi intermediates and the Golgi complex by enhancing recruitment of coatomers to pre-Golgi intermediates^45–47^. Taken together with our results, these findings point to a possibility that relative abundance of Rab2-GTP at the pre-Golgi intermediates may influence the maturation of pre-Golgi intermediates and thereby the efficiency of anterograde trafficking of glycosyltransferases between pre-Golgi intermediates and the cis-Golgi compartments.

Because Golgin45 CC2 and CC3 appear to contain two independent binding sites for Rab2-GTP (Fig. 7) and Golgin45 likely forms a parallel homodimer like many other Golgin tethers, it is possible that a Golgin45 homodimer can bind up to four Rab2-GTP molecules, simultaneously. Based on this logic, it would be plausible to consider that TNKS1-mediated degradation of Golgin45 at the *cis*- and *medial*-Golgi compartments can cause a dynamic shift of available Rab2-GTP pool between the Golgi and the pre-Golgi intermediates, leading to reduced trafficking between pre-Golgi intermediates and the Golgi, although this hypothesis needs to be investigated in a future study.

In general, dynamic fusion/fission of the Golgi ribbon structure is considered to be essential for efficient large cargo transport across the Golgi complex^20,29,48,49^. Since large oversize cargo trafficking showed no significant changes in response to increased Golgin45 protein level in our study (Fig. 4h), we suspect that Rab2 may play a different role in lateral fusion of Golgi stacks during large cargo transport.

Lastly, but importantly, our results showed that Golgin45 provides a major docking site for TNKS1 targeting to the Golgi and that the majority of TNKS1 localizes to the Golgi apparatus in interphase cells. Since TNKS1 was originally identified as a nuclear protein that is involved in telomere maintenance along with a negative regulator of telomere length, TRF1 (TTAGGG repeat binding factor 1)^1,4,50,51^, Golgin45-mediated targeting of TNKS1 to the Golgi and Cdc2-dependent modulation of their interaction raise a possibility that Golgin45/TNKS1 complex may also be a part of Golgi mitotic checkpoint^52,53^ or plays a role in cellular senescence-related glycosylation changes at the Golgi^54,55^, which requires a future study.

In summary, our findings show that (i) TNKS1 targeting to the Golgi membranes is dependent on Golgin45; (ii) TNKS1-mediated regulation of Golgin45 seems to have a profound influence on glycosyltransferase trafficking and protein glycosylation at the Golgi; (iii) Golgin45-Rab2-GTP interaction plays an important role in this process.

## Materials and methods

### Reagents and antibodies

All common reagents were purchased from Sigma-Aldrich, unless otherwise mentioned. XAV939, MG132 and nocodazole were purchased from Selleck. The following antibodies were used: mouse monoclonal anti-Tankyrase1/2 (1:1000 for WB and 1:100 for IF, sc-365897, Santa Cruz), mouse monoclonal anti-Golgin45 (1:1000 for WB, MA5-27126, Thermo), rabbit polyclonal anti-Golgin45 (1:300 for IF, PA5-30714, Thermo), rabbit polyclonal anti-GRASP55 (1:3000 for WB and 1:500 for IF, 10598-1-AP, Proteintech), mouse monoclonal anti-GRASP55 (1:500 for IF, ab211532, Abcam), anti-GM130 (1:2000 for WB and 1:1000 for IF, ab52649, Abcam), anti-Golgin97 (1:1000 for WB and 1:300 for IF, 13192 S, Cell Signaling Technology), anti-mCherry (1:3000 for WB, ab167453, Abcam), anti-PARG (1:1000 for WB, ALX-202-045-UC01, ENZO), anti-Axin1 (1:1000 for WB, 16541-1-AP, Proteintech), anti-Giantin (1:1000 for WB, ab174655, Abcam), anti-Golgin160 (1:1000 for WB, ab96080, Abcam), anti-p230 (1:1000 for WB, 611280, BD bioscience), anti-GMAP210 (1:1000 for WB, 26456-1-AP, Proteintech), anti-TMF1 (1:1000 for WB, ab151702, Abcam), anti-GPP130 (1:1000 for WB, 923801, Biolegend), anti-Golgin84 (1:1000 for WB, HPA00099, Sigma-Aldrich), anti-ACBD3 (1:1000 for WB, HPA015594, Sigma-Aldrich), anti-p115 (1:1000 for WB, 13509-1-AP, Proteintech), anti-GOLPH3 (1:1000 for WB, 19112-1-AP, Proteintech), anti-GRASP65 (1:1000 for WB, ab174834, Abcam), anti-PI4KB (1:1000 for WB, 611816, BD bioscience), anti-GBF1 (1:1000 for WB, ab86071, Abcam), anti-ARFGEF1 (1:1000 for WB, ab183747, Abcam), anti-ARFGEF2 (1:1000 for WB, ab236951, Abcam), anti-ARFGAP1 (1:3000 for WB, ab204405, Abcam), anti-ARFGAP2 (1:1000 for WB, ab133768, Abcam), anti-ARFGAP3 (1:3000 for WB, 15293-1-AP, Proteintech), anti-ARF1 (1:1000 for WB, 10790-1-AP, Proteintech), anti-β-COP (1:3000 for WB, ab2899, Abcam), anti-γ-COP (1:1000 for WB, sc-393615, Santa cruz), anti-COG4 (1:3000 for WB, ab154795, Abcam), anti-COG8 (1:1000 for WB, 12661-1-AP, Proteintech), anti-Syntaxin 5 (1:3000 for WB, 110053, Synaptic Systems), anti-Syntaxin 6 (1:1000 for WB, 110062, Synaptic Systems), anti-GS27 (1:1000 for WB, 12095-1-AP, Proteintech), anti-GAPDH (1:5000 for WB, KC-5G5, Kangchen Bio-tech), HRP-conjugated anti-His-tag (1:5000 for WB, HRP66005, Proteintech), anti-poly(ADP-ribose) (1:200 for IP, 1020/N, Tulip Biolabs), anti-SNAP tag (1:1000 for WB, P9310S, NEB), anti-collagen IV (1:300 for IF, ab6586, Abcam), HRP-conjugated Wheat Germ Agglutinin (1:5000 for WB, WGA-HRP, 29073, biotium). Anti-Rabbit Alexa Fluor 488 (1:500 for IF, A21441), Alexa Fluor 568 (1:500 for IF, A10042), Alexa Fluor 647 (1:500 for IF, A21245) and anti-Mouse Alexa Fluor 488 (1:500 for IF, A21200), Alexa Fluor 568 (1:500 for IF, A10037), Alexa Fluor 647 (1:500 for IF, A21236) for Immunofluorescence were obtained from ThermoFisher. All siRNA oligos were were custom designed by Shanghai GenePharma, China. The target sequences were as following: Human Golgin45 #1 (gggaacagtttcgtcaaga); Human Golgin45 #2 (GATTCCATCAACAGTTGAA); Human GRASP55 siRNA: GGCAUUGGAUAUGGUUAUU; human tankyrase-1 (GCATGGAGCTTGTGTTAAT); human tankyrase-2 (GGAAAGACGTAGTTGAATA). The sequence of the non-targeting control siRNA was UUCUCCGAACGUGUCACGU.

### Cell culture and and transfection

HeLa (ATCC, CCL-2) and COS7 (Stem Cell Bank, Chinese Academy of Sciences) cells were grown in DMEM supplemented with 10% FBS (Thermo) at 37 °C. Transfection of DNA constructs and siRNAs was performed using Lipofectamine 2000 and RNAiMAX (ThermoFisher), respectively, according to the manufacturer’s instructions. For DNA expression, cells were transfected 24 h before Co-IP experiments and 18 h for IF experiments. For siRNA knockdown, cells were transfected 72 h before experiments.

### Golgin45 stable knockdown cell lines

The stable knockdown of Golgin45 was achieved by infecting target cells using lentivirus expression of Golgin45 shRNA (GCAGAGCTAGCATTAACAAAT). The lentivirus was packaged and commercially provided by Shanghai GenePharma, China. Cells were infected with the lentivirus expressing Golgin45 shRNA using Polybrene (Sigma) overnight. Two days after infection, the cells were cultured in puromycin (0.3–1 μg/ml, ThermoFisher) for 2 weeks.

### Co-immunoprecipitation (Co-IP) and Immunoblotting

For Co-IP experiments, total lysates were prepared using lysis buffer (25 mM HEPES, pH 7.4, 150 mM NaCl, 1% NP-40, 1x protease inhibitor cocktail (Roche)). Subsequently, the total lysates were passed through a syringe needle (15 times) and then incubated at 4 °C with end-over-end agitation for 1.5 h. The lysates were then cleared by centrifugation at 15,000 × *g* for 20 min. The supernatants were incubated with anti-RFP agarose beads (M165-8, MBL life science) for 4 h at 4 °C with end-over-end agitation. The beads were washed two times with ice-cold lysis buffer and one time with PBS. Proteins were eluted by boiling in 2x SDS running buffer and subjected to SDS-PAGE for immunoblotting.

For immunoblotting, proteins were separated by SDS-PAGE (Genscript) and transferred onto nitrocellulose membranes (Amersham). Membranes were blocked with 3% bovine serum albumin (BSA) and then probed with specific primary antibodies and then with peroxidase-conjugated secondary antibodies (Jackson ImmunoResearch). The bands were visualized with chemiluminescence (Clarity Western ECL Substrate, Bio-Rad) and imaged by a ChemiDoc Touch imaging system (Bio-Rad). Representative blots are shown from several experiments.

For detection of glycoprotein bands on blots using WGA-HRP. The blots were blocked with 3% bovine serum albumin (BSA) for 1 h and were probed with WGA-HRP in PBS-T for 1 h at room temperature. The blots were washed 5 times for 10 min each with PBS. After five washes, the blots were visualized with chemiluminescence (Clarity Western ECL Substrate, Bio-Rad) and imaged by a ChemiDoc Touch imaging system (Bio-Rad).

### Immunofluorescence staining

Cells grown on glass coverslips (72230-01, Electron Microscopy Sciences) in 24-well plates or glass bottom 24-well plates (P24-1.5H-N, Cellvis) were fixed for 10 min with 4% paraformaldehyde (PFA), permeabilized in permeabilization Buffer (0.3% Igepal CA-630, 0.05% Triton-X 100, 0.1% IgG-free BSA in PBS) for 5 min, and blocked in blocking buffer (0.05% Igepal CA-630, 0.05% Triton-X 100, 5% normal goat serum in PBS) for 60 min. Primary and secondary antibodies were applied in blocking buffer for 1 h. The nucleus was stained with Hoechst-33342 (sc-200908, Santa Cruz Biotechnology). Cells were washed three times with wash buffer (0.05% Igepal CA-630, 0.05% Triton-X 100, 0.2% IgG-free BSA in PBS) and twice with PBS. Coverslips were mounted using ProLong Gold Antifade Reagent (ThermoFisher). Dip coverslip in diH2O before mounting to prevent salt contamination. Images were acquired with a Zeiss LSM880 confocal microscope using a 63x Apochromat oil-immersion objective. 3D-structured illumination microscopy (SIM) imaging was acquired using Nikon N-SIM microscope.

### Cell Surface Biotinylation

Cells grown in 6-well plates to 80% confluency were transfected using 1 μg plasmid DNA and 2 μl Lipofectamine 2000 for 24 h. For anterograde transport of TfR-FM4-SNAP (a kind gift by Andreas M. Ernst), transfected cells were treated with 5 μM XAV939 for 12 h and 50 µg/ml cycloheximide for 2 h, prior to induction of synchronized protein secretion by treatment with d/d-solubilizer drug for the indicated time points. During the biotinylation procedure, all reagents and cell cultures were kept on ice. Cells were washed twice in ice-cold PBS and subsequently incubated in 1 ml/well of a 1 mM Sulfo-NHS-LC-Biotin (APExBIO) in PBS solution for 30 min on ice. The cells were then washed in quenching buffer (100 mM glycine in PBS), and incubated in 1 ml/well of quenching buffer for 15 min on ice. The cells were washed twice with PBS and then lysed in 300 µl of lysis buffer (50 mM Tris, pH 7.4, 150 mM NaCl, 0.1% SDS, 1% (v/v) Triton X-100, 0.5% (w/v) deoxycholate, and protease inhibitor cocktail (Roche)). Lysates were incubated for 20 min on ice and sonicated for 20 seconds. Finally, the lysate was centrifuged at 4 °C for 10 min at 15000× *g*. Supernatants were incubated with 40 μl of Streptavidin Agarose beads (S1638, Sigma Aldrich) with constant rocking for 1 h at RT. The samples were washed three times with PBS, then eluted with 2x SDS-sample buffer for 10 min at 95 °C and used for Western blot.

### Collagen IV transport and secretion assay

For monitoring endogenous Collagen IV transport in COS7 cells, WT cells or cells pretreated with XAV939 for 9 h were subjected to folding block (40 °C, 3 h) without ascorbate. A traffic pulse of Collagen IV was induced by shifting cells to 32 °C in the presence of 100 mg/mL ascorbate and 50 μg/ml Cycloheximide for the indicated times.

Collagen IV secretion in COS7 cells were assessed by Human Collagen IV ELISA Kit (FineTest), according to the manufacturer’s protocols.

### Cdc2 Kinase assay

The in vitro Cdc2 kinase assays were carried out using mitotic cell lysates, biotinylated synthetic peptides and a commercial Cdc2 Kinase assay kit, according to the manufacturer’s instruction (Cell Signaling Technology).

### Fluorescence recovery after photobleaching (FRAP)

For FRAP experiments, HeLa cells transfected with GalT1-GFP were treated with DMSO or XAV939. The cells were imaged with a ×63 objective on a Zeiss LSM 880 confocal microscope in an atmosphere of 5% CO_2_ at 37 °C. Part of the Golgi was bleached using a single laser pulse. Images were acquired every 10 s for 5 min. Fluorescence values in the bleached and an adjacent non-bleached area were measured using Fiji. Fluorescence recovery is represented as the ratio of the bleached to the adjacent areas, normalized to the pre-bleach and immediate post-bleach values. Experiments were repeated twice and the graph was plotted using average values from these experiments (±SEM).

For FRAP experiments with nocodazole treatment, HeLa WT cells, cells treated with Golgin45 siRNA, cells pretreated with Golgin45 siRNA and rescue with siRNA-resistant mutant of mCherry-Golgin45 WT or V214/273 A, or cells treated with XAV939 grown in 35 mm glass bottom dishes (Cellvis) were transfected with GalT1-EGFP for 16 h, then the cells were treated 10 μM nocodazole for 3 h to scatter the Golgi. The cells were imaged with a 63x objective on a Zeiss LSM 880 confocal microscope in an atmosphere of 5% CO_2_ at 37 °C. The boxed area indicated in the figures was photobleached. Images were acquired every 30 seconds for 20 min after the bleach. Fluorescence values in the bleached and an adjacent non-bleached area were measured. Fluorescence recovery is represented as the ratio of the bleached to the adjacent areas, normalized to the pre-bleach and immediate post-bleach values.

### Protein expression and purification

The DNA coding sequence of TNKS1-Arc1 (183–335aa), TNKS1-Arc2 (336–489aa), TNKS1-Arc3 (490–653aa), TNKS1-Arc4 (654–806aa), TNKS1-Arc5(807–961aa) and Golgin-45(cc#1-3; 120-276aa) were inserted in a pET24b plasmid for the expression with C-terminal 6xHis tag.

The DNA coding sequence of Rab1a, Rab2a, Rab6a, Rab11a, Rab33b and Golgin45 (1-70aa) were inserted in pGEX-6p-1 plasmid for the expression of GST fusion proteins.

Plasmids were transformed to Escherichia coli BL21 (DE3) cells and induced with 0.3 mM isopropyl β-D-1-thiogalactopyranoside (IPTG) for 12 h at 16 °C. GST fusion proteins were affinity purified with Glutathione HiCap Matrix (Qiagen). His tag proteins were purified with Ni-NTA Agarose (Qiagen).

### Biolayer interferometry (BLI) Assays

BLI experiments were performed with Octet RED96 instrument (ForteBio). GST fusion proteins were loaded to GST biosensors at 10ug/ml for 300 s. Binding to TNKS1-Arc1 domain was monitored for 600 s at 25 °C in 25 mM Hepes pH 7.4, 150 mM NaOAc, 0.2% Trition, 1 mM DTT. The dissociation steps were monitored for 300 s. Six concentrations of analytic proteins were assayed (0.16, 0.31, 0.63, 1.25, 2.5, and 4.56 μM). Affinities were determined by fitting the concentration dependence of the experimental steady-state signals, using the Octet RED data analysis v10 software (ForteBio).

### GST-Pulldown Assays

For each pulldown assay, 150 μg GST fusion proteins were immobilized by incubating with 40ul of 50% (vol/vol) Glutathione-Sepharose 4B beads (GE Healthcare) at 4 °C for 1 h. Beads were washed three times and incubated with 0.1 μM prey protein in binding buffer (25 mM Hepes pH 7.4, 150 mM NaOAc, 0.2% Trition and 1 mM DTT) at 4 °C for 2 h.

For GST-Rab GTPase pulldown assays, the GST-Rab proteins immobilized on Glutathione-Sepharose 4B (GE Healthcare) were incubated in 25 mM Hepes pH 7.4, 1 mM EDTA for 30 min at 25 °C. Beads were washed for three times and incubated with Guanosine 5′-diphosphate sodium salt (GDP; 100 μM; Sigma-Aldrich)or Guanosine 5′-O-(3-thiotriphosphate) tetralithium salt (GTPγS; 100 μM; Sigma-Aldrich) in 25 mM Hepes pH 7.4, 150 mM NaOAc, 25 mM Mg(OAc)2 for 60 min at 25 °C. Beads were washed three times and incubated with 0.1 μM prey protein in binding buffer (25 mM Hepes pH 7.4, 150 mM NaOAc, 25 mM Mg(OAc)2, 0.2% Trition and 1 mM DTT) at 4 °C for 2 h.

After pulldown incubation, beads were washed for 4 times and 40 μl 2x SDS loading buffer were added. After boiling the samples were aspirated from the beads and volumes of samples were finalized to 80 μl. 2% Input and 20% samples were loaded for Western Blot assays.

### Yeast two-hybrid assay

The yeast two-hybrid assay was carried out using the Matchmaker gold yeast two-hybrid system (Clontech), according to the manufacturer’s instructions. Briefly, human Golgin45 (CC#1-3) and its mutants were subcloned in the GAL4 activation domain vector pGADT7 (Prey). The GTP or GDP-locked Rab2 mutants (Rab2-Q65L and Rab2-S20N) were subcloned into the Gal4 DNA binding domain vector, pGBKT7 (Bait). The Y2HGold yeast strains co-transformed with bait/prey pair were spreaded onto selective medium lacking leucine and tryptophan (SD/-Leu/-Trp, DDO, Clontech). The physical binding of bait and prey was identified by colony selection in selective medium lacking adenine, leucine, tryptophan and histidine (SD/-Ade/-Leu/-Trp/-His, Clontech) supplemented with X-α-Gal and Aureobasidin A (QDO/ X-α-Gal/AbA).

### N-linked glycan preparation for analysis by mass spectrometry

The tryptic digested samples were resuspended in 5% acetic acid and desalted by BakerBond spetm Octadecyl (C18) disposable extraction column (J. T. Baker). The samples were heated at 100 °C for 5 min to inactivate trypsin and dried completely by SpeedVac. Subsequently, dried samples were res-dissolved in 29 μL of 1x buffer solution, pH 5.0. Next, each sample was incubated with 1 μL peptide-N-glycosidase F (PNGase F, New England, Biolabs, Inc) for 20 h at 37 °C. The N-linked glycans released were purified and eluted from peptides mixture by reverse phase cartridge (Sep-Pak C_18_) with a 5% acetic acid solution. The samples were dried by SpeedVac and finally permethylated. The isotopically labeled (^13^C) maltotetraose as internal standard was spiked in a constant amount (5 pmol) to each sample to improve the precision of mass spectrometry analysis.

### Analysis of N-linked glycan by mass spectrometry

The samples were sodiated by 1 mM NaOH in 80% MeOH then directly infused onto a 30 µm fused silica emitter (New Objective). The detection of N-linked glycans was performed on the Q Exactive™ Plus Orbitrap Mass Spectrometer (Thermo Scientific) furnished with a Nanospray Flex Ion Source for direct infusion at 0.5 µL/min flow rate. The full MS spectra were obtained for relative quantitative analysis with 30 s data acquisition time at 600 to 2000 Da mass range.

### Preparation of an isotope-labeled maltotetraose as an internal standard

In this study, the method of internal standard was used to improve the precision of the relative quantification for the N-linked glycans from the HeLa cells. Firstly, maltotetraose (Sigma, DP4) was selected for the internal standard (IS), followed by the permethylation with ^13^C-iodomethane (^13^CH_3_I). The permethylated maltotetraose whose mass increased by14.0476 Da (ΔM) to 899.4785 Da (mono) served as the internal standard characterized by a specific mass on the mass spectrum. The 5 pmol of the isotopically labeled maltotetraose dissolved in methanol was spiked to each sample prepared from HeLa cells.

### Statistics and reproducibility

Pearson coefficient and line intensity of 3D-SIM images were analyzed by Fiji software. Results are displayed as mean ± SD (standard deviation) of results from each experiment or dataset, as indicated in figure legends. All statistical tests were performed using Student’s *t* tests or ANOVA (GraphPad, Prism 8.0). Significance values are assigned in specific experiments. N (number of individual experiments) is noted in the figure legends. A two-tailed paired *t* test type was used to determine *p* values between two conditions of the FRAP experiments. *N* (number of individual experiments) is noted in the figure legends. All figures show the data from independent samples or otherwise are indicated in the figure legend as representative data from independent experiments.

### Reporting summary

Further information on research design is available in the Nature Research Reporting Summary linked to this article.

## Supplementary information

Supplementary Information

Description of Additional Supplementary Files

Supplementary Data 1

Reporting Summary

## Data Availability

All data generated or analyzed during this study are included in this published article and its supplementary information files. Source data for figures can be found in Supplementary Data 1. Original blot images are shown in Supplementary Fig. 4. All relevant data relating to this manuscript are available from the corresponding author on reasonable request.
