# Supplementary material for: Tankyrase-1-mediated degradation of Golgin45 regulates glycosyltransferase trafficking and protein glycosylation in Rab2-GTP-dependent manner
Source: Commun Biol. 2021 Dec 7;4:1370. doi: 10.1038/s42003-021-02899-0 (PMC8651787; doi:10.1038/s42003-021-02899-0)

supplementary Fig.1

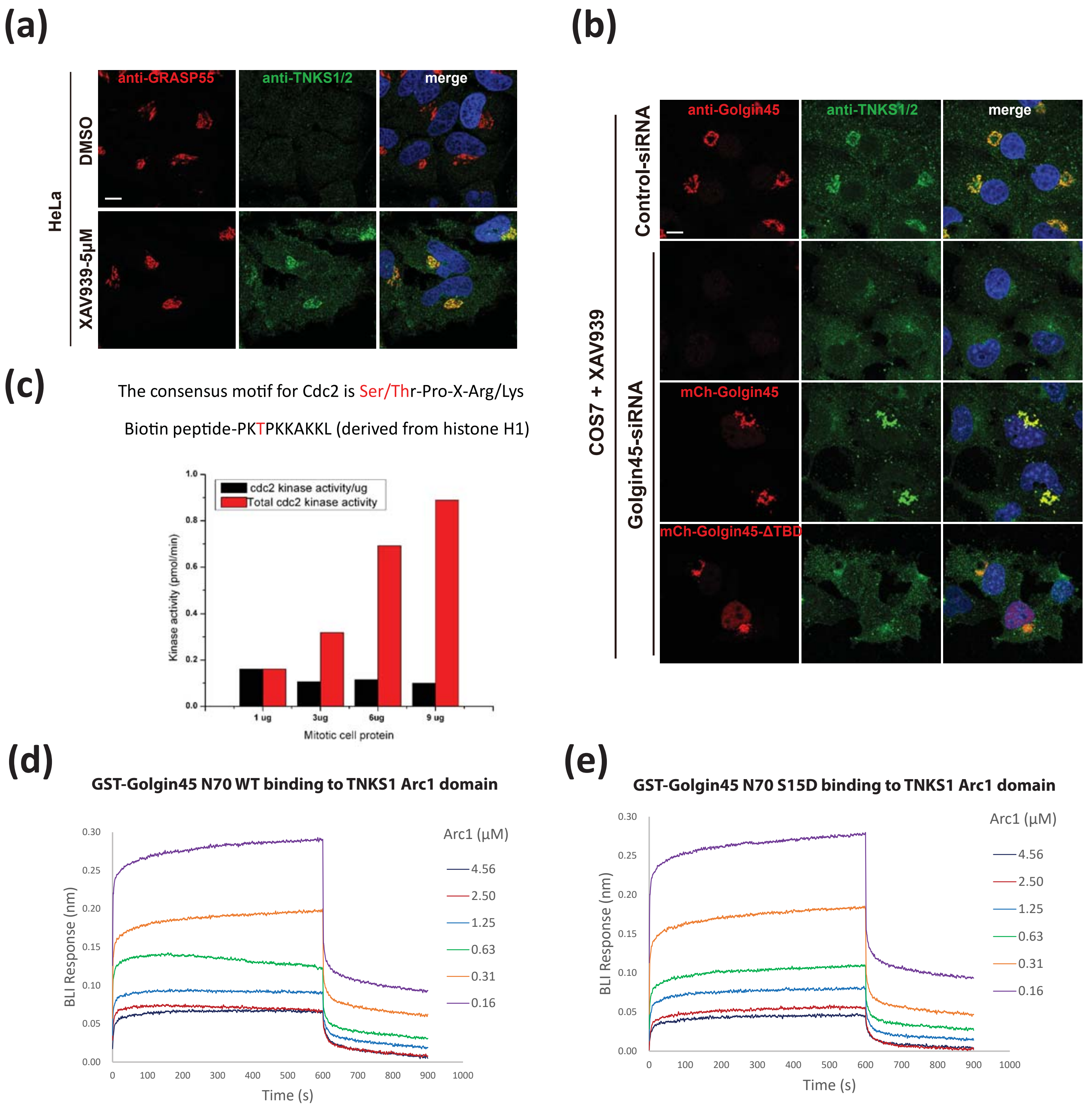

supplementary Figure 1

(a) Inhibition of TNKS1 activity by XAV939 increases Golgi signals of TNKS1/2 under confocal microscope.HeLa cells were treated with either DMSO or XAV939 for 12 hours, followed by fixing and staining with antibodies against endogenous TNKS1/2 and GRASP55 (as a Golgi marker). scale bar, 10 mm; (b) confocal micrographs of COS7 cells showed that Golgin45 knockdown disrupts the Golgi localization of TNKS1/2, which could be restored by exogenous expression of RNAi-resistant mCherry-Golgin45, but not by Golgin45-ΔTBD. Scale bar, 10 mm; (c) in vitro Cdc2 activity assay using increasing amount of mitotic cytosol from HeLa cells. We used Histone-1 synthetic peptide (TPKK) as a substrate; (d)-(e) Binding profiles of the Golgin45-N70-WT and S15D to the TNKS1-ARC1 domain measured by biolayer interferometry in an Octet RED96 instrument. GST fusion proteins were loaded to GST biosensors and tested for binding with gradient concentrations of the soluble TNKS1-ARC1 domain. Affinities were determined by fitting the concentration dependence of the experimental steady state signals, using the Octet RED data analysis v10 software.

supplementary Figure 2

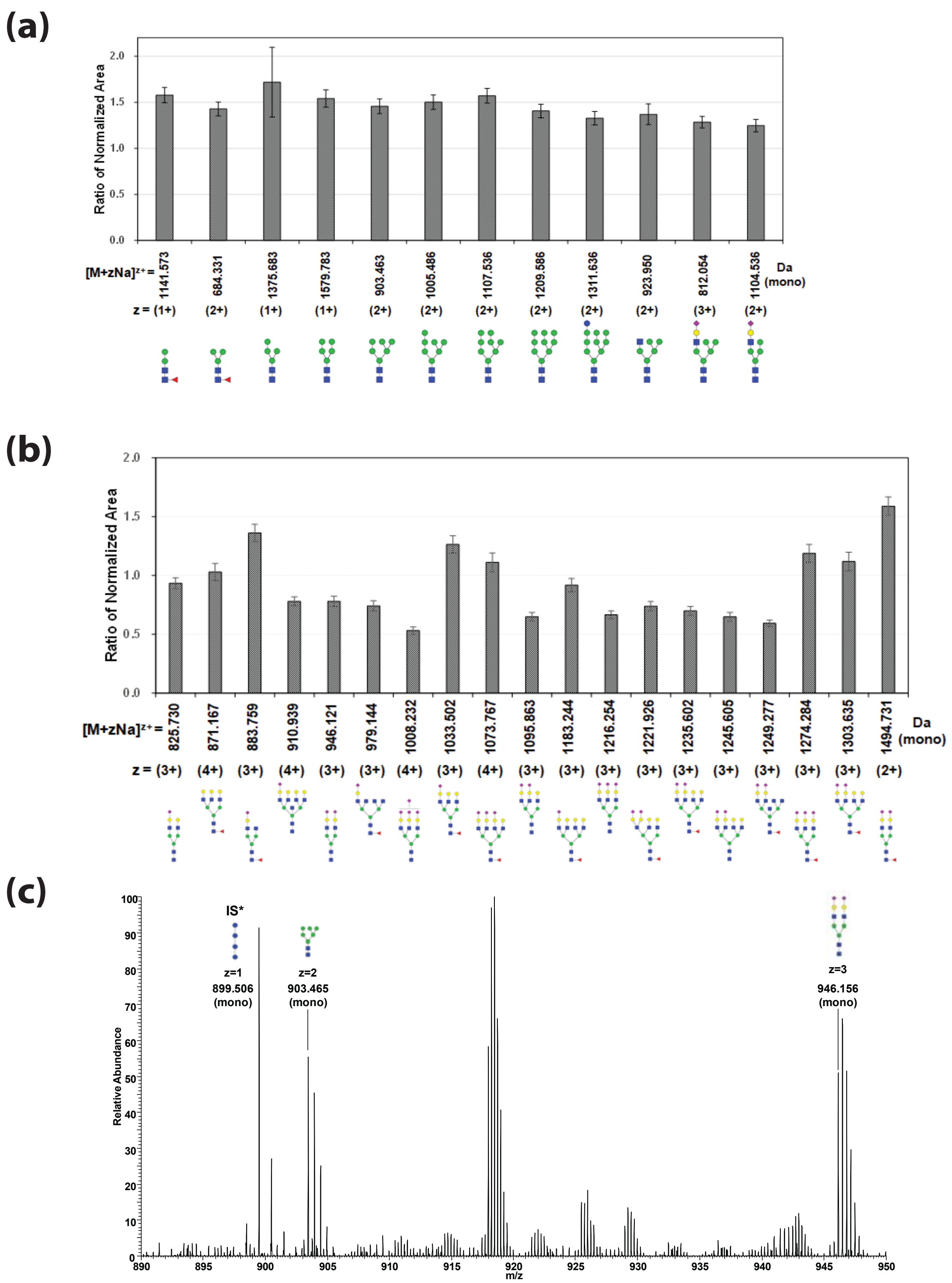

Supplementary Figure 2
(a) Ratio of normalized XAV peak area to normalized DMSO peak area for high mannose and hybrid N-linked glycans. (b) Ratio of normalized XAV peak area to normalized DMSO peak area for complex N-linked glycans. Low abundance glycans, eight non-sialylated complex N-linked glycans with a normalized area of less than 0.09, were excluded from the histogram. (c) Comparision of full mass spectrum of isotope-labeled internal standard, high mannose, and complex N-linked glycans for normalization.

supplementary Figure 3

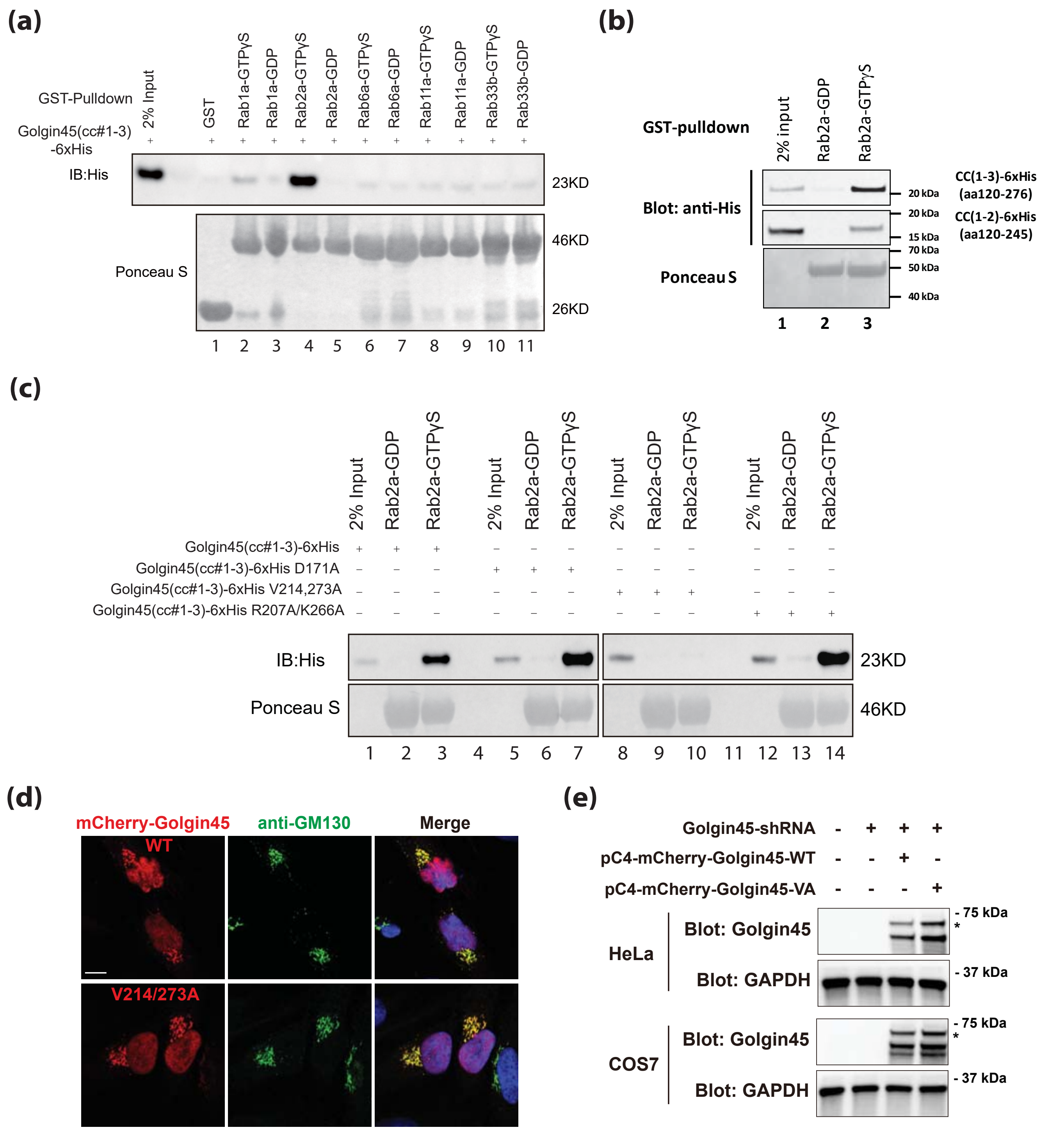

Supplementary Figure 3
(a) Recombinant Golgin45 CC domains selectively interact with Rab-2-GTP. GST pull-down assays were performed using purified recombinant Golgin45 CC#1-3 domains and GST-Rab1a, 2a, 6a, 11a, 33b. (b) GST pull-down assays were performed using purified recombinant Golgin45 CC#1-3 or CC#1-2 domains and GST-Rab2a pre-loaded with either GDP or GTPgS. (c) GST pull-down assays confirming that V214/273A mutation selectively inhibit Golgin45 interaction with Rab2-GTP. GST pull-down assays were performed using purified recombinant Golgin45 CC#1-3 WT or mutants (D171A, V214/273A or R204/K266A) domains and GST-Rab2a. (d) V214/273A mutation shows no influence on the Golgi localization of mCherry-Golgin45. Scale bar, 10mm. (e) Western blots showing the loading control (GAPDH) and expression of mCherry-Golgin45 WT or mCherry-Golgin45 V214/273 mutant in cells treated with Golgin45 RNAi oligos for 48 hours for lectin blots shown in Figure 7D.

Supplementary Figure 4
Unprocessed images of all blots

Unprocessed western blots in main figures. Some blots were cut into several pieces and incubated with different antibodies.

Unprocessed blots of Figure 1B

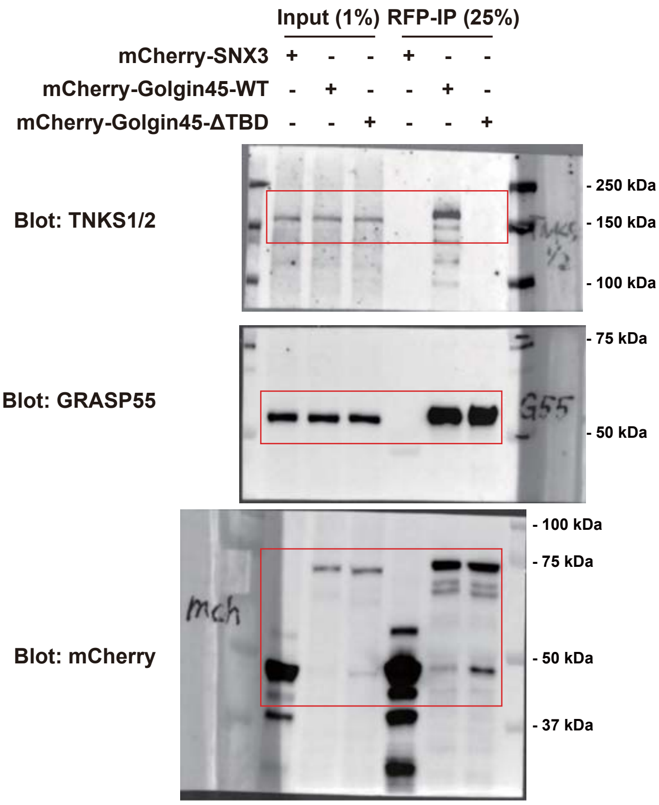

Unprocessed blots of Figure 1C

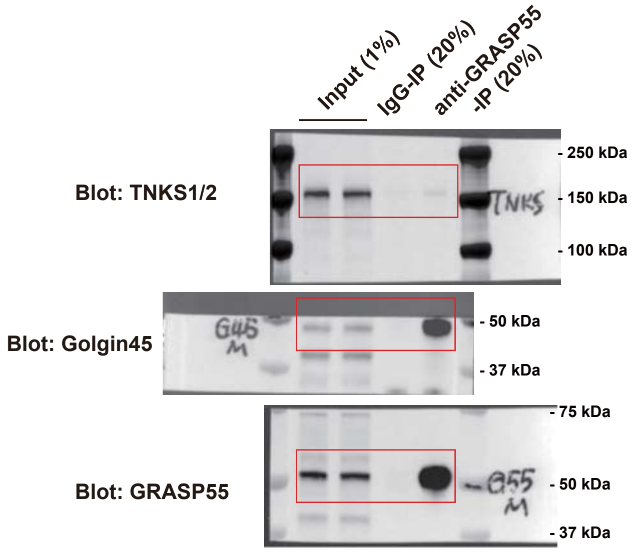

Unprocessed blots of Figure 1D

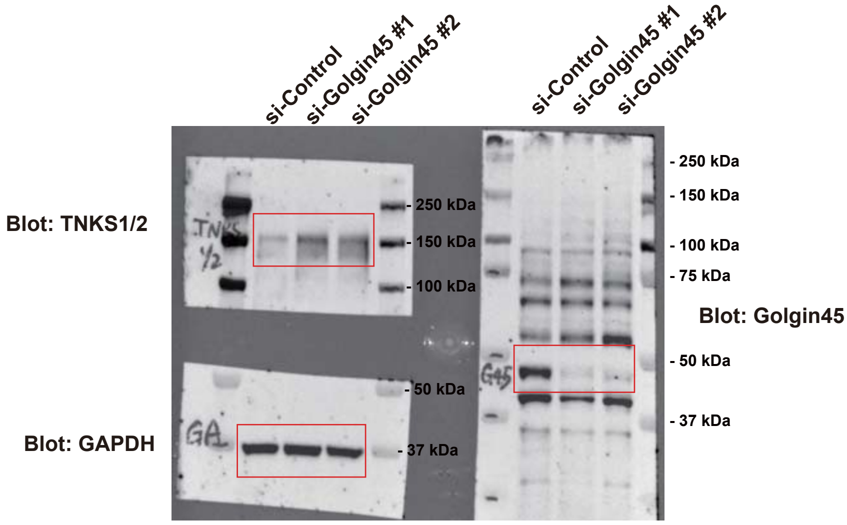

Unprocessed blots of Figure 1G

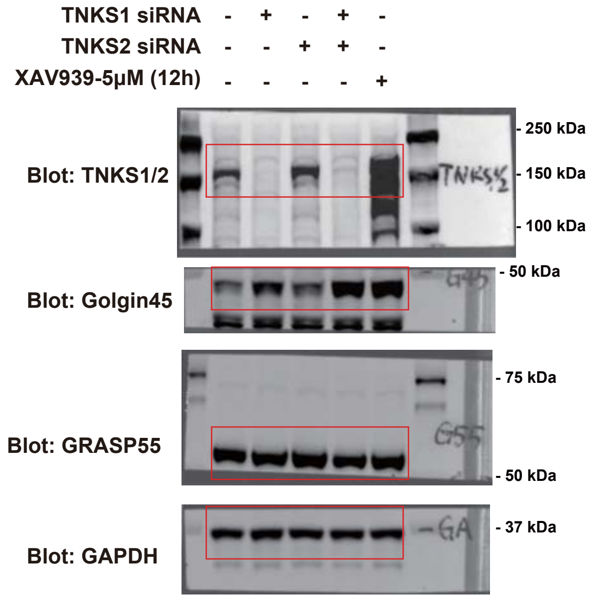

Unprocessed blots of Figure 2A

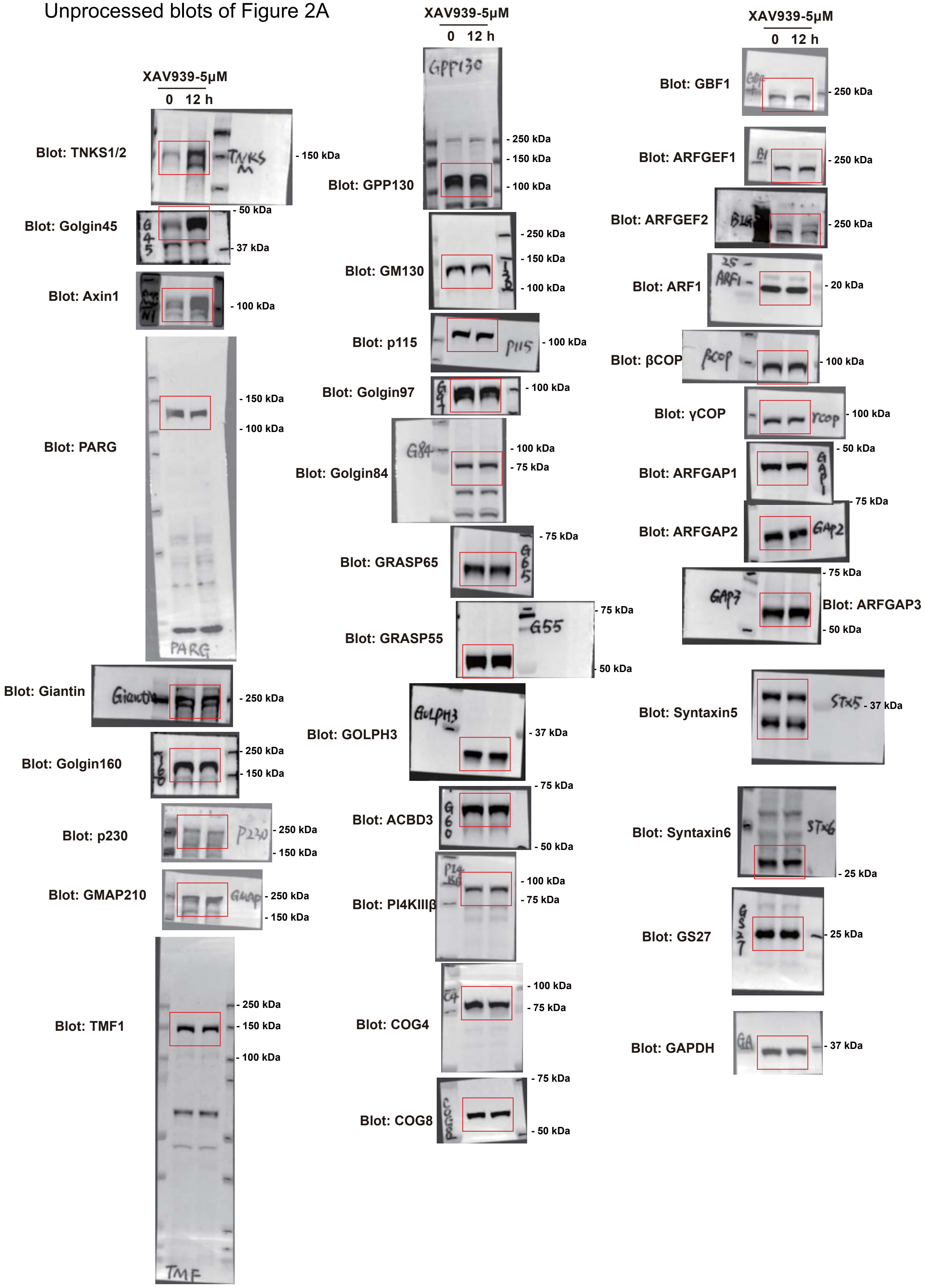

Unprocessed blots of Figure 2B

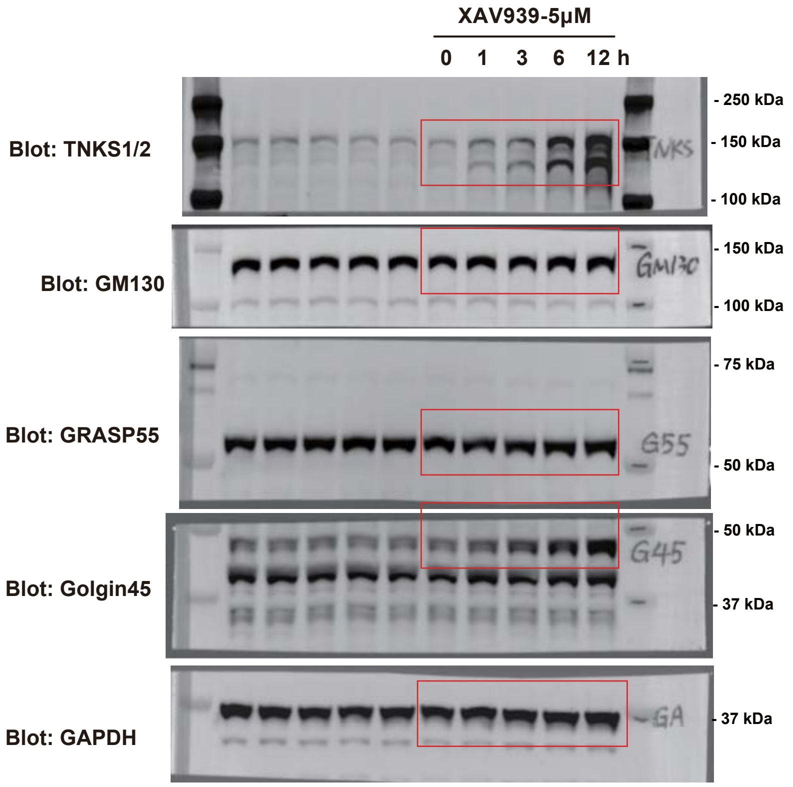

Unprocessed blots of Figure 2C

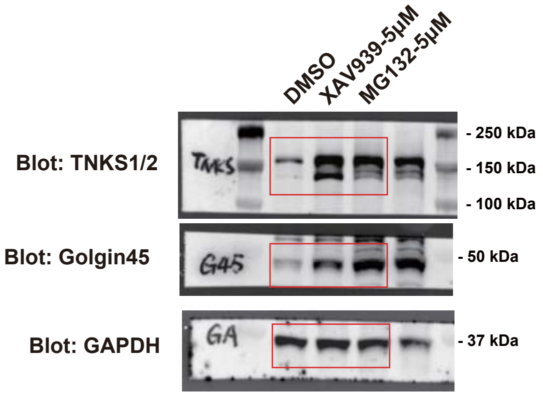

Unprocessed blots of Figure 2D

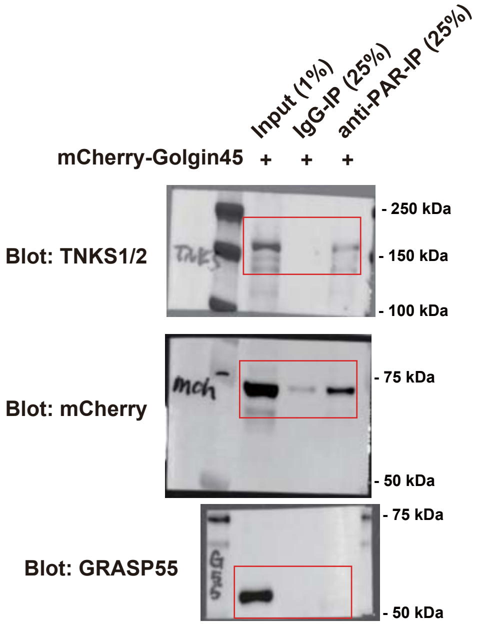

Unprocessed blots of Figure 2E

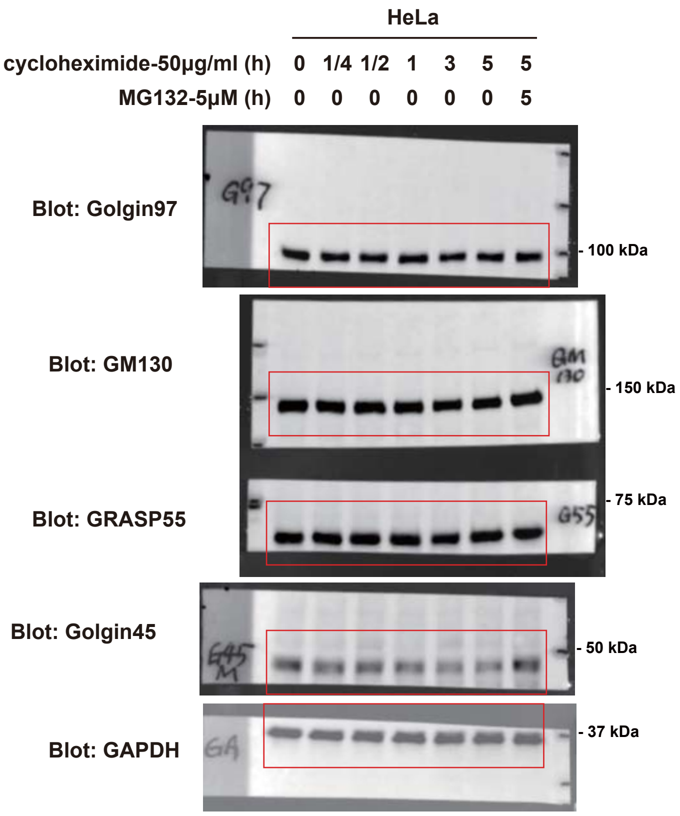

Unprocessed blots of Figure 3B

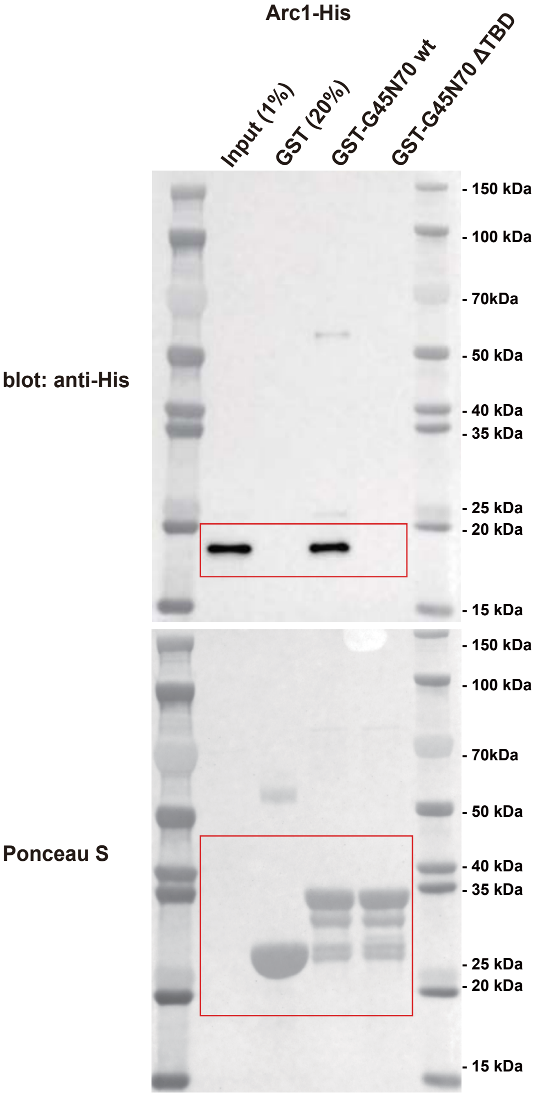

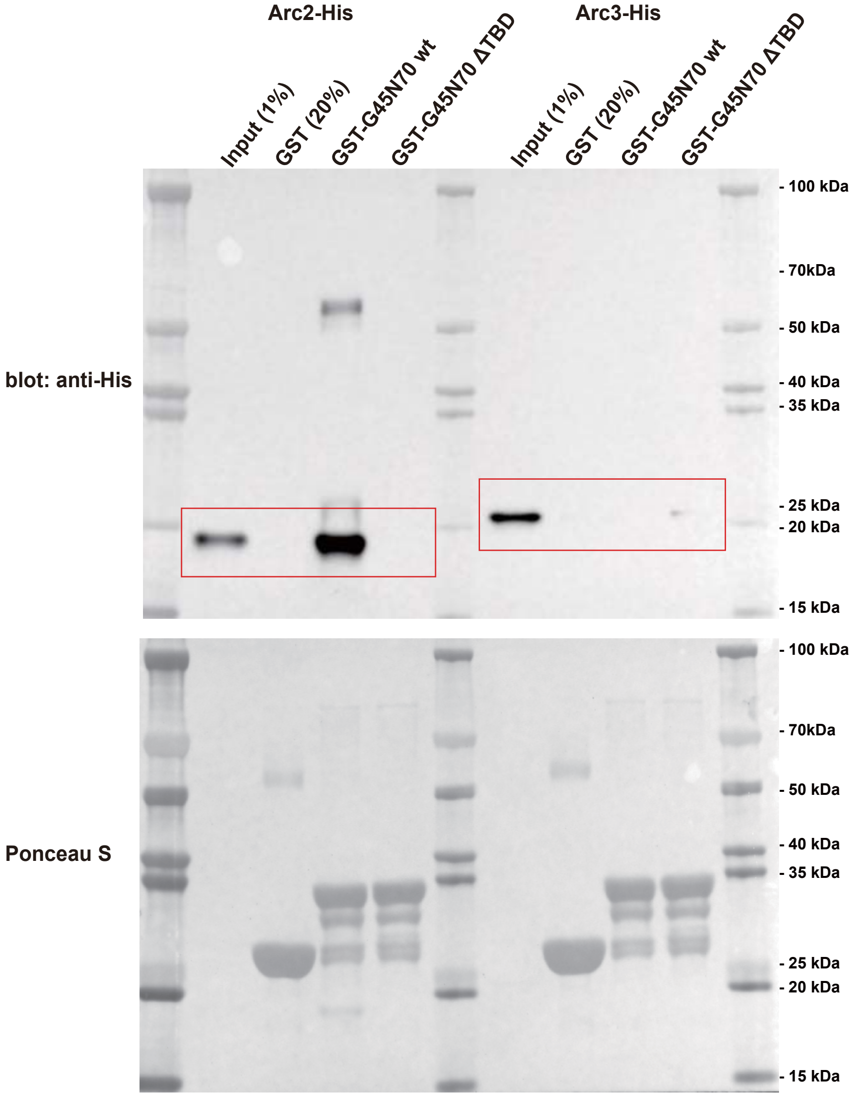

Unprocessed blots of Figure 3B

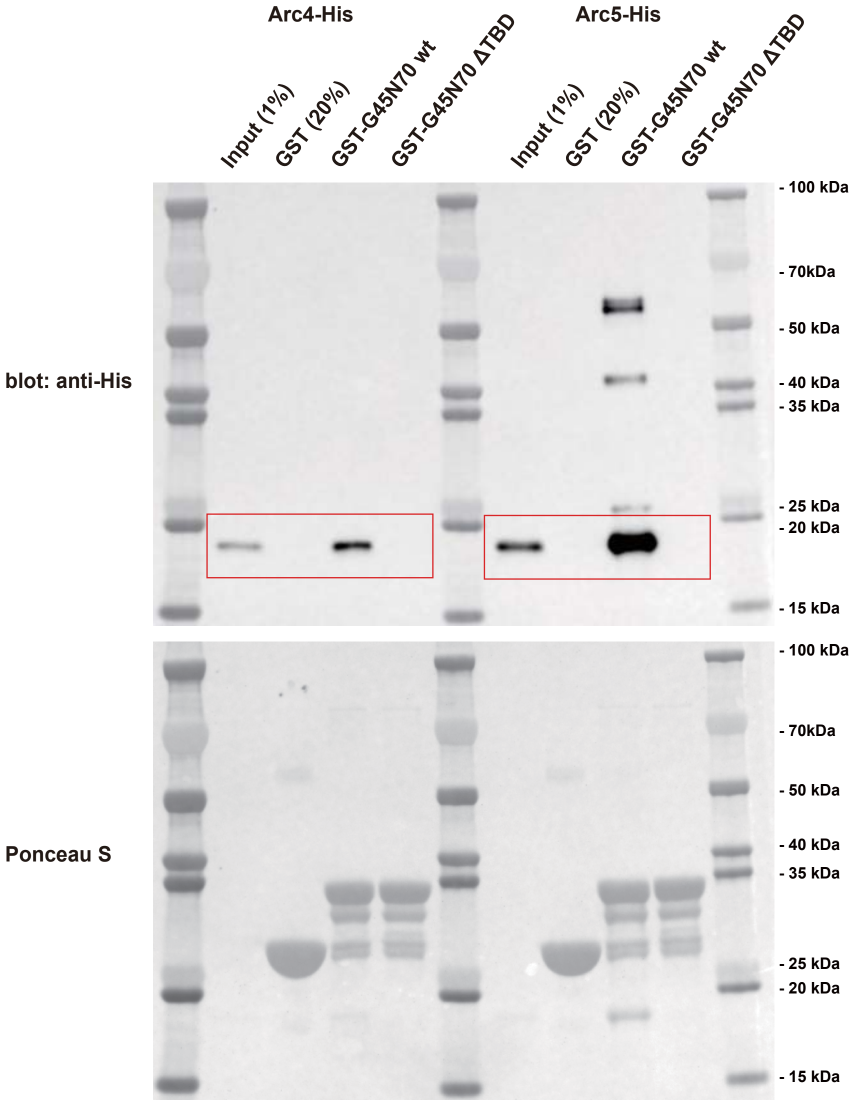

Unprocessed blots of Figure 3E

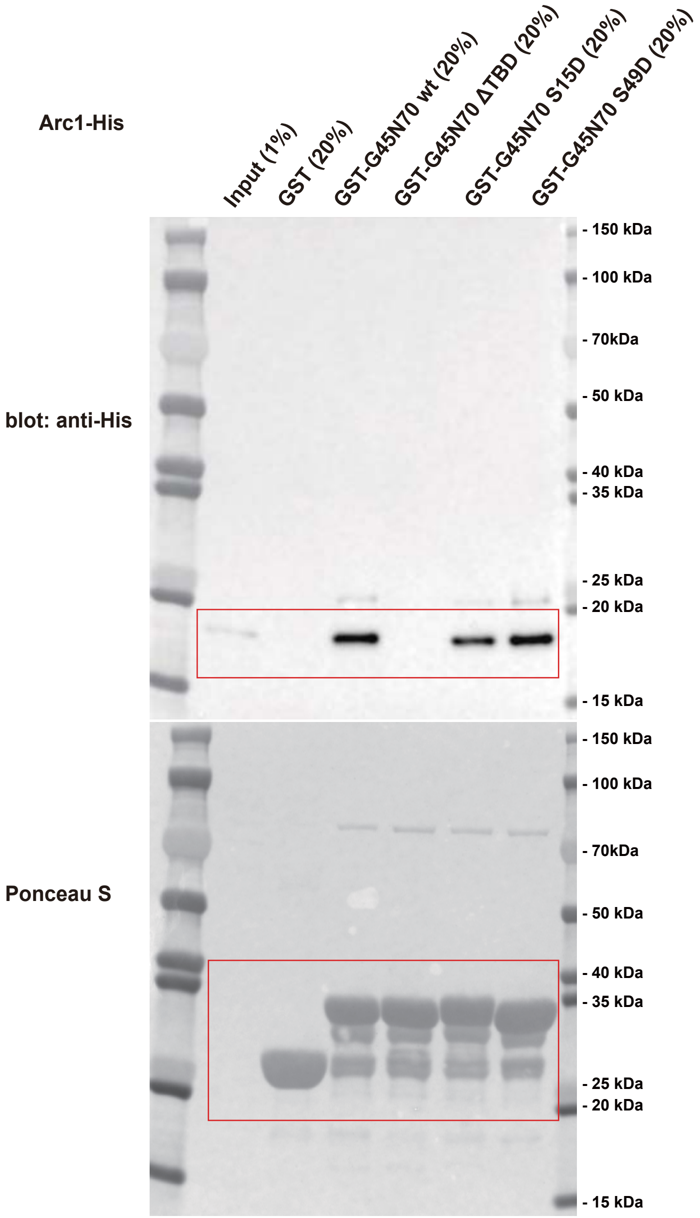

Unprocessed blots of Figure 4A

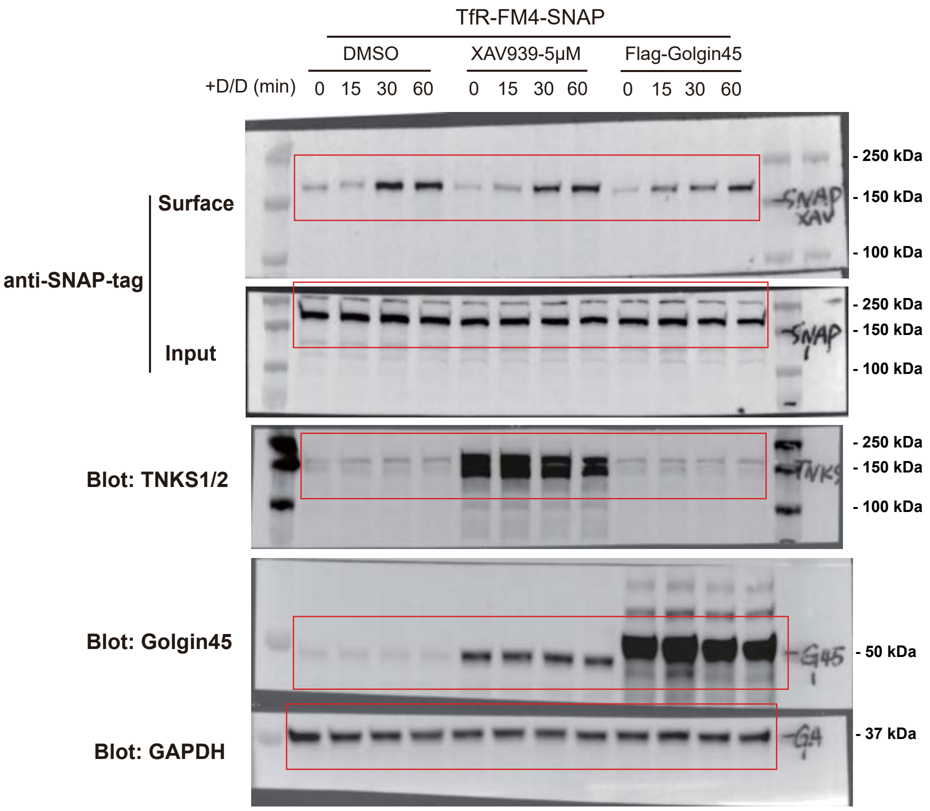

Unprocessed blots of Figure 4B

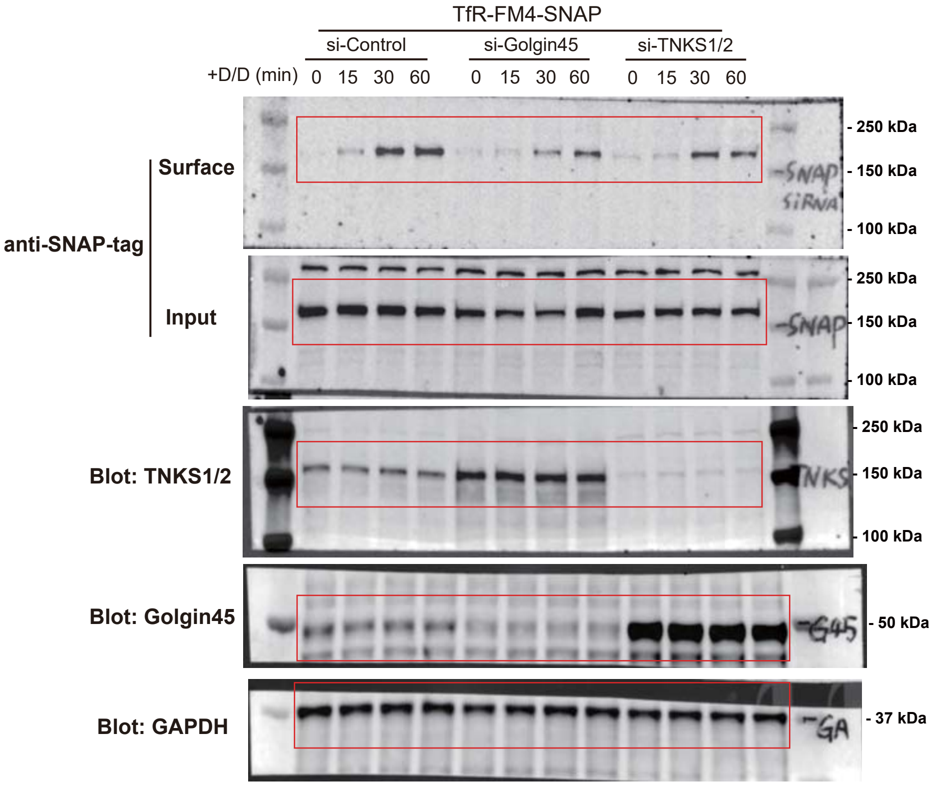

Unprocessed blots of Figure 4I

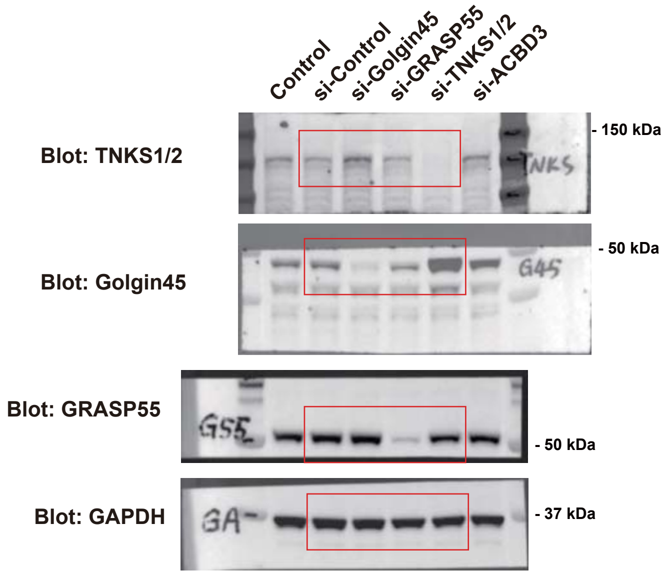

Unprocessed blots of Figure 5G

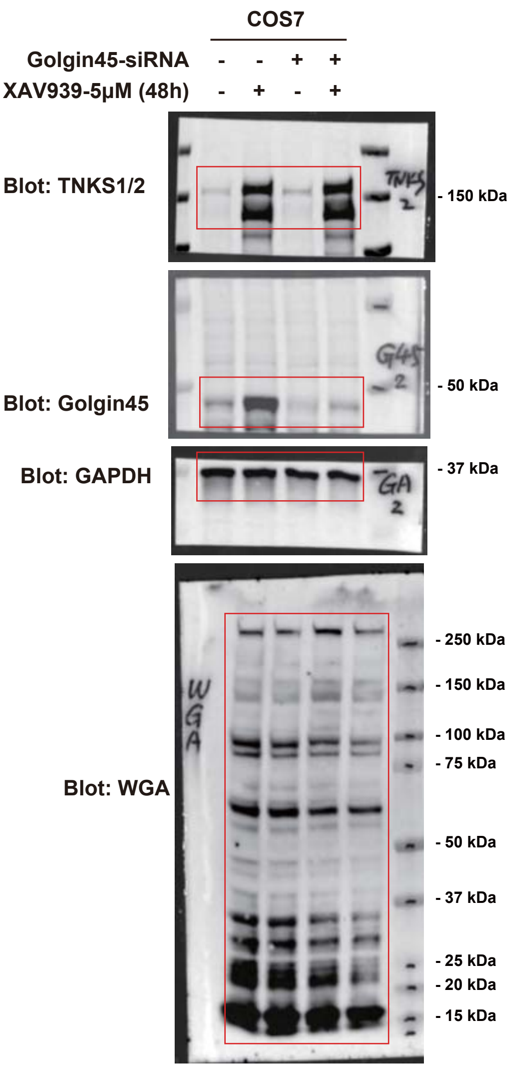

Unprocessed blots of Figure 6G

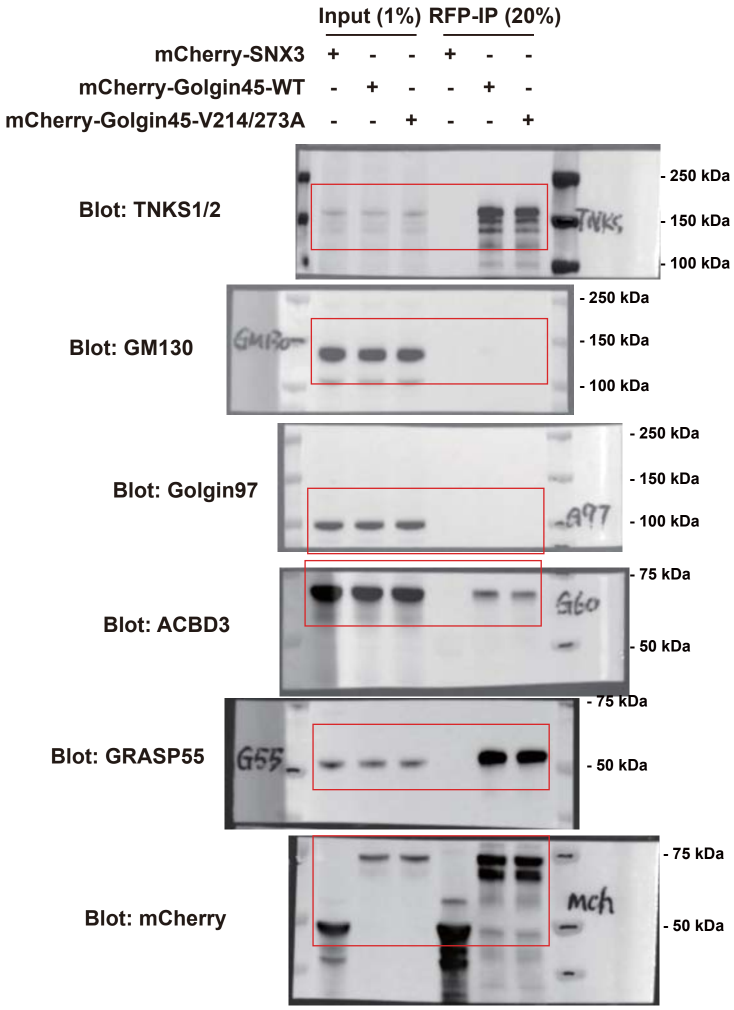

Unprocessed blots of Figure 7C

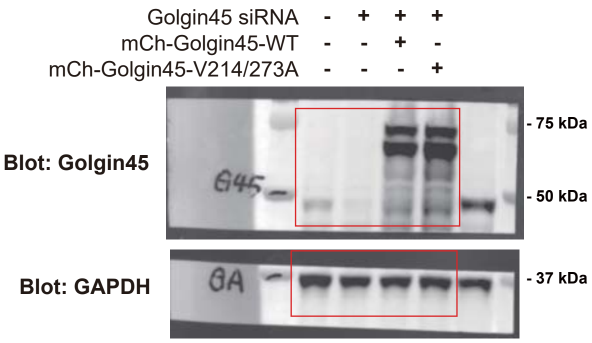

Unprocessed blots of Figure 7D and supplementary Figure 3D

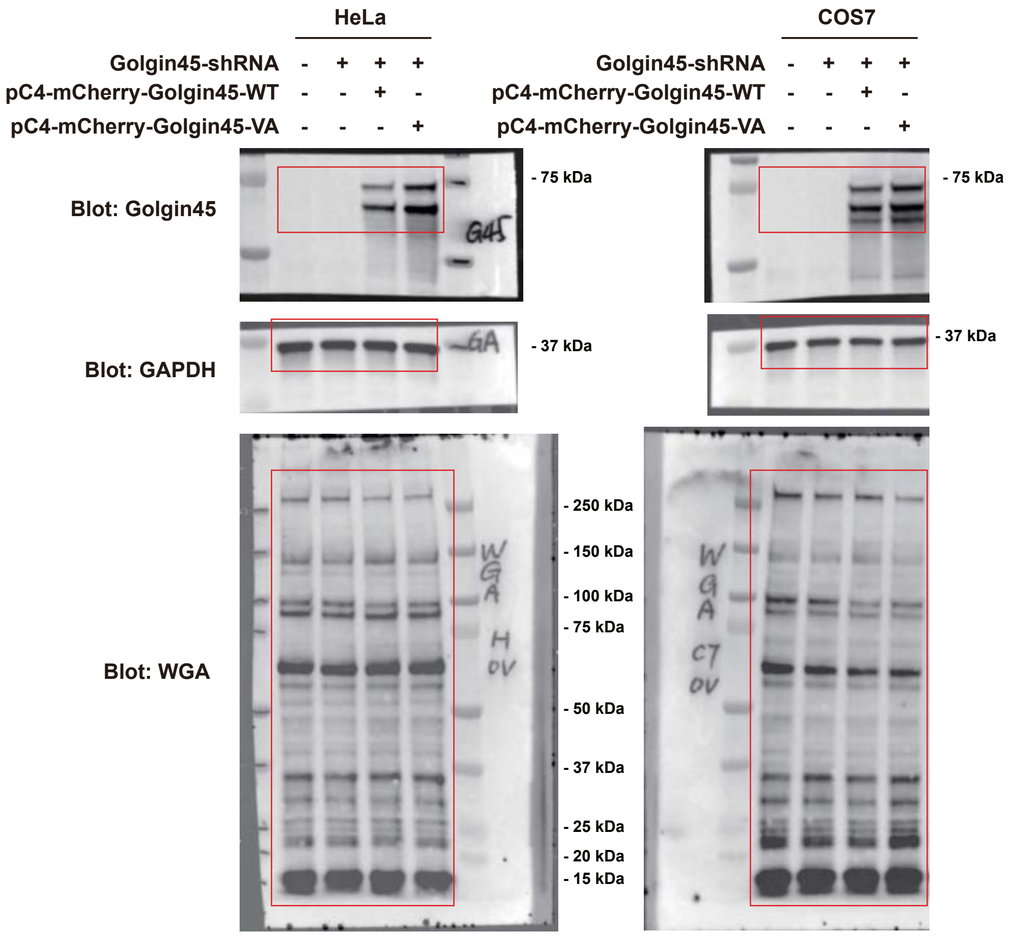

Unprocessed blots of Figure S3A

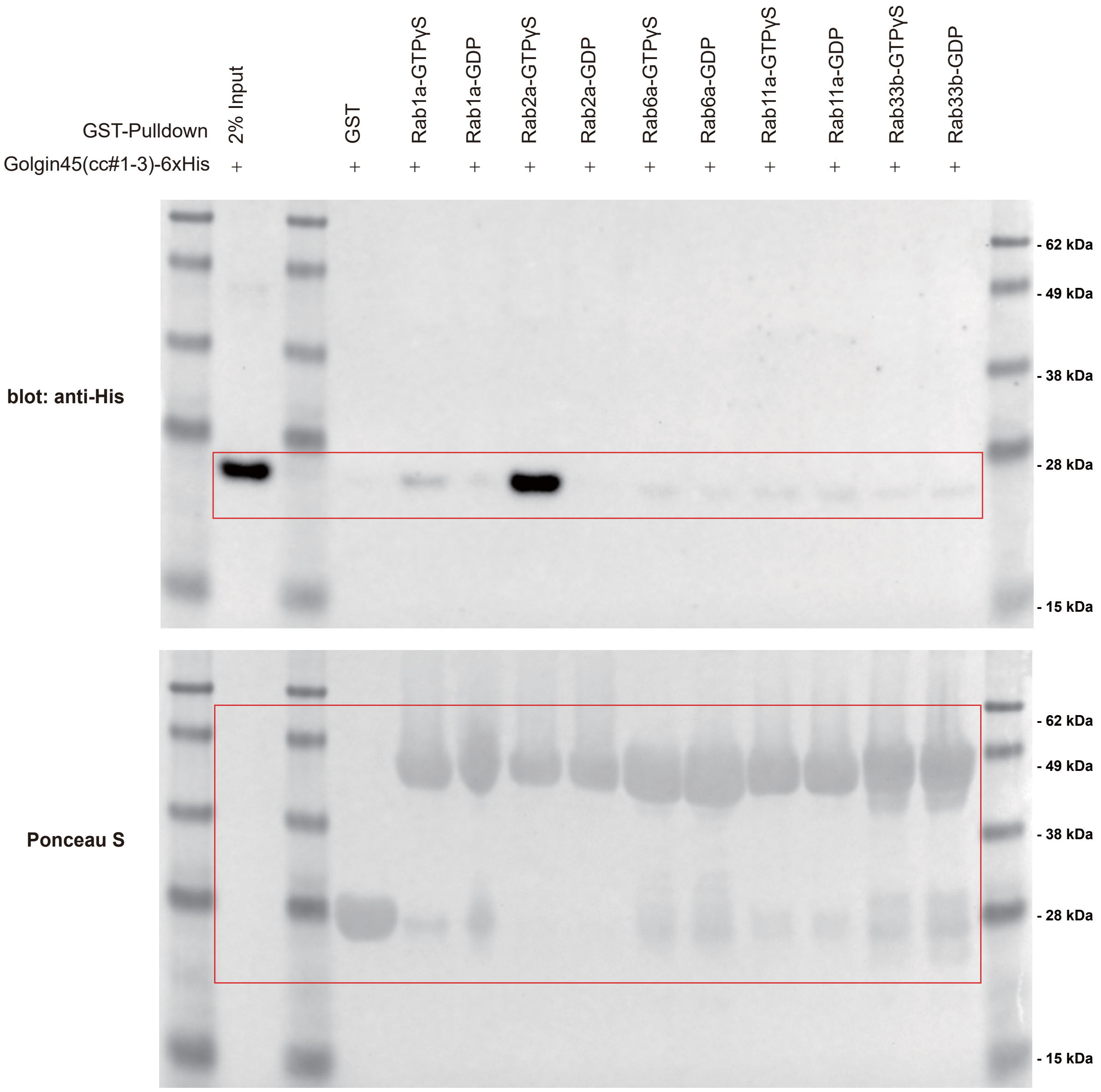

Unprocessed blots of Figure S3B

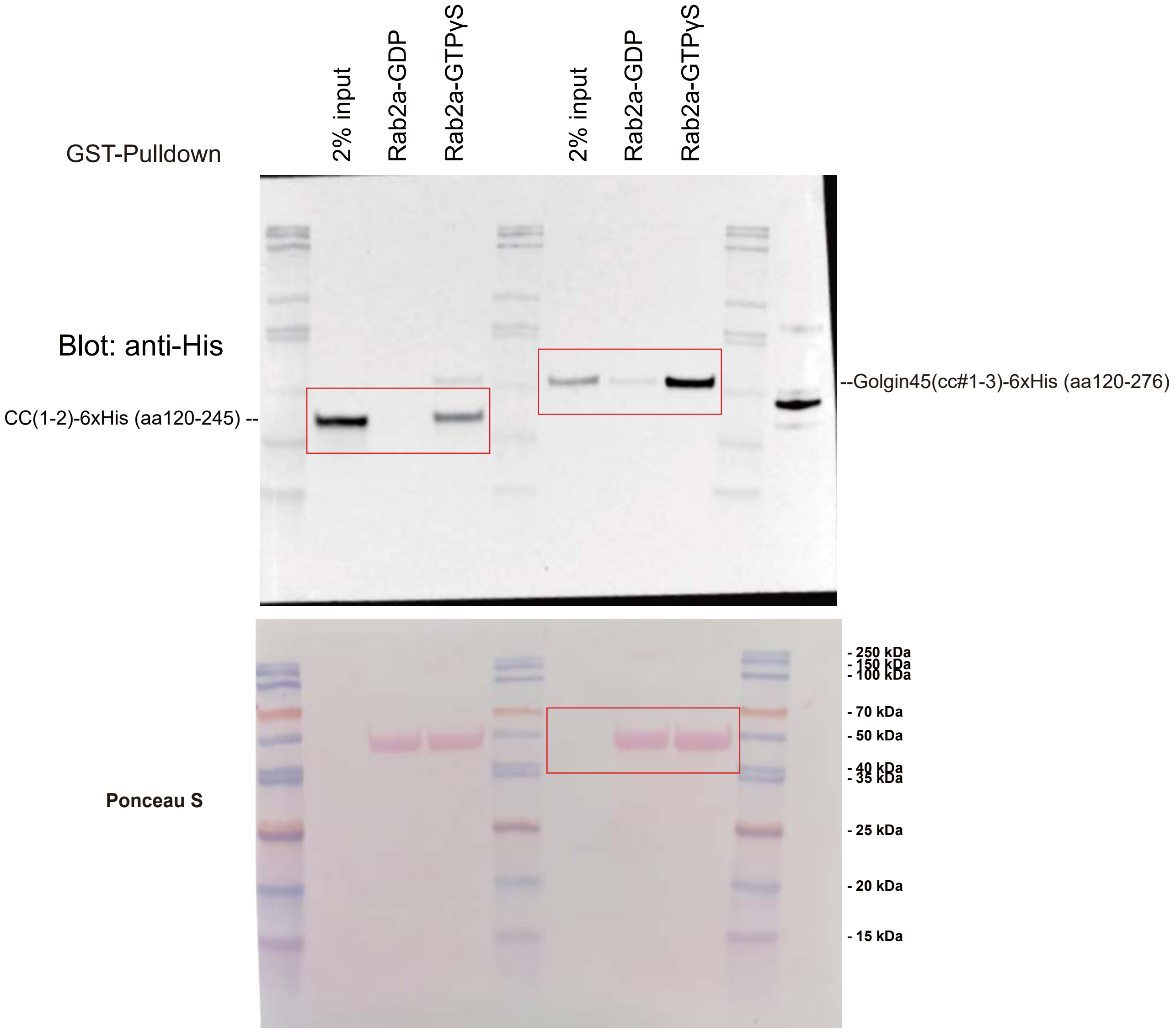

Unprocessed blots of Figure S3C

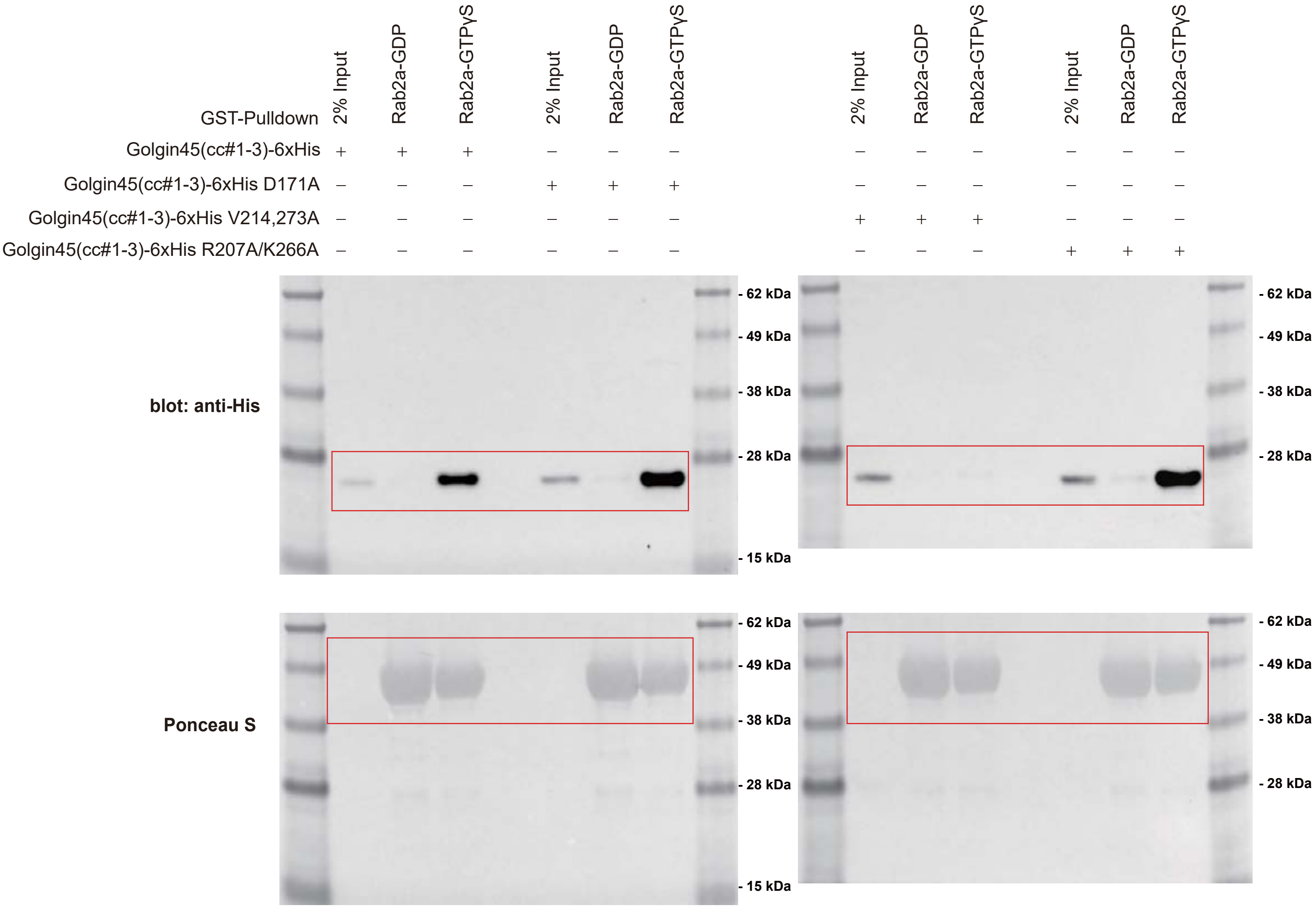

Supplement: Supplementary file 1 — Supplementary Information [file 42003_2021_2899_MOESM1_ESM.pdf]
