# Supplementary material for: Tankyrase-1-mediated degradation of Golgin45 regulates glycosyltransferase trafficking and protein glycosylation in Rab2-GTP-dependent manner
Source: Commun Biol. 2021 Dec 7;4:1370. doi: 10.1038/s42003-021-02899-0 (PMC8651787; doi:10.1038/s42003-021-02899-0)
Supplement: Supplementary file 2 — Description of Additional Supplementary Files [file 42003_2021_2899_MOESM2_ESM.pdf]

Description of Additional Supplementary Files

File name: Supplementary Data 1

Description: Source data for all graphs in the main figures.
