# Supplementary material for: Tankyrase-1-mediated degradation of Golgin45 regulates glycosyltransferase trafficking and protein glycosylation in Rab2-GTP-dependent manner
Source: Commun Biol. 2021 Dec 7;4:1370. doi: 10.1038/s42003-021-02899-0 (PMC8651787; doi:10.1038/s42003-021-02899-0)
Supplement: Supplementary file 4 — Reporting Summary [file 42003_2021_2899_MOESM4_ESM.pdf]

nature research

Corresponding author(s): INTAEK LEE

Last updated by author(s): Oct 25, 2021

Reporting Summary

Nature Research wishes to improve the reproducibility of the work that we publish. This form provides structure for consistency and transparency in reporting. For further information on Nature Research policies, see our Editorial Policies and the Editorial Policy Checklist.

Statistics

For all statistical analyses, confirm that the following items are present in the figure legend, table legend, main text, or Methods section.

n/a	Confirmed	
☐	☒	The exact sample size (*n*) for each experimental group/condition, given as a discrete number and unit of measurement
☐	☒	A statement on whether measurements were taken from distinct samples or whether the same sample was measured repeatedly
☐	☒	The statistical test(s) used AND whether they are one- or two-sided<br>*Only common tests should be described solely by name; describe more complex techniques in the Methods section.*
☒	☐	A description of all covariates tested
☒	☐	A description of any assumptions or corrections, such as tests of normality and adjustment for multiple comparisons
☐	☒	A full description of the statistical parameters including central tendency (e.g. means) or other basic estimates (e.g. regression coefficient) AND variation (e.g. standard deviation) or associated estimates of uncertainty (e.g. confidence intervals)
☐	☒	For null hypothesis testing, the test statistic (e.g. F, t, r) with confidence intervals, effect sizes, degrees of freedom and P value noted<br>*Give P values as exact values whenever suitable.*
☒	☐	For Bayesian analysis, information on the choice of priors and Markov chain Monte Carlo settings
☒	☐	For hierarchical and complex designs, identification of the appropriate level for tests and full reporting of outcomes
☐	☒	Estimates of effect sizes (e.g. Cohen's d, Pearson's r), indicating how they were calculated

Our web collection on statistics for biologists contains articles on many of the points above.

Software and code

Policy information about availability of computer code

Data collection	ZEN software (Carl Zeiss), NIS-Elements AR (Nikon), Image Lab software (Bio-Rad), Octet RED v10 software (ForteBio).
Data analysis	Prism 8.0 (Graphpad), Fiji (ImageJ).

For manuscripts utilizing custom algorithms or software that are central to the research but not yet described in published literature, software must be made available to editors and reviewers. We strongly encourage code deposition in a community repository (e.g. GitHub). See the Nature Research guidelines for submitting code & software for further information.

Data

Policy information about availability of data

All manuscripts must include a data availability statement. This statement should provide the following information, where applicable:

- Accession codes, unique identifiers, or web links for publicly available datasets
- A list of figures that have associated raw data
- A description of any restrictions on data availability

Data are available from corresponding author upon request.

Field-specific reporting

Life sciences study design

All studies must disclose on these points even when the disclosure is negative.

Sample size	No sample size calculation was performed in this study. Relevant literature study and search guided us to determine the group numbers in each experiments. For quantification of immunofluorescence data, at least 10 different cells in each experiments were included for data analysis. At least three independent experiments were performed to obtained data and to determine the statistical significance.
Data exclusions	No data were excluded from the study.
Replication	All attempts for replication were successful. At least three independent applications were carried out for each qualification analysis.
Randomization	Samples were picked randomly to statistical analysis.
Blinding	No animals or human research participants are involved in this study.

Reporting for specific materials, systems and methods

We require information from authors about some types of materials, experimental systems and methods used in many studies. Here, indicate whether each material, system or method listed is relevant to your study. If you are not sure if a list item applies to your research, read the appropriate section before selecting a response.

Materials & experimental systems

n/a	Involved in the study	
[ ]	[x]	Antibodies
[ ]	[x]	Eukaryotic cell lines
[x]	[ ]	Palaeontology and archaeology
[x]	[ ]	Animals and other organisms
[x]	[ ]	Human research participants
[x]	[ ]	Clinical data
[x]	[ ]	Dual use research of concern

Methods

n/a	Involved in the study	
[x]	[ ]	ChIP-seq
[x]	[ ]	Flow cytometry
[x]	[ ]	MRI-based neuroimaging

Antibodies

Antibodies used	mouse monoclonal anti-Tankyrase1/2 (1:1000 for WB and 1:100 for IF, sc-365897, Santa Cruz), mouse monoclonal anti-Golgin45 (1:1000 for WB, MA5-27126, Thermo), rabbit polyclonal anti-Golgin45 (1:300 for IF, PA5-30714, Thermo), rabbit polyclonal anti-GRASP55 (1:3000 for WB and 1:500 for IF, 10598-1-AP, Proteintech), mouse monoclonal anti-GRASP55 (1:500 for IF, ab211532, Abcam), anti-GM130 (1:2000 for WB and 1:1000 for IF, 610822ab52649, BD bioscienceAbcam), anti-Golgin97 (1:1000 for WB and 1:300 for IF, A2127013192S, ThermoCell Signaling Technology), anti-Flag (F1804, Sigma-Aldrich), anti-mCherry (1:3000 for WB, ab167453, Abcam), anti-PARG (1:1000 for WB, ALX-202-045-UC01, ENZO), anti-Axin1 (1:1000 for WB, 16541-1-AP, Proteintech), anti-Giantin (1:1000 for WB, ab174655, Abcam), anti-Golgin160 (1:1000 for WB, ab96080, Abcam), anti-p230 (1:1000 for WB, 611280, BD bioscience), anti-GMAP210 (1:1000 for WB, 26456-1-AP, Proteintech), anti-TMF1 (1:1000 for WB, ab151702, Abcam), anti-GPP130 (1:1000 for WB, 923801, Biolegend), anti-Golgin84 (1:1000 for WB, HPA00099, Sigma-Aldrich), anti-ACBD3 (1:1000 for WB, HPA015594, Sigma-Aldrich), anti-p115 (1:1000 for WB, 13509-1-AP, Proteintech), anti-GOLPH3 (1:1000 for WB, 19112-1-AP, Proteintech), anti-GRASP65 (1:1000 for WB, ab174834, Abcam), anti-PI4KB (1:1000 for WB, 611816, BD bioscience), anti-GBF1 (1:1000 for WB, ab86071, Abcam), anti-ARFGEF1 (1:1000 for WB, ab183747, Abcam), anti-ARFGEF2 (1:1000 for WB, ab236951, Abcam), anti-ARFGAP1 (1:3000 for WB, ab204405, Abcam), anti-ARFGAP2 (1:1000 for WB, ab133768, Abcam), anti-ARFGAP3 (1:3000 for WB, 15293-1-AP, Proteintech), anti-ARF1 (1:1000 for WB, 10790-1-AP, Proteintech), anti-β-COP (1:3000 for WB, ab2899, Abcam), anti-γ-COP (1:1000 for WB, sc-393615, Santa cruz), anti-COG4 (1:3000 for WB, ab154795, Abcam), anti-COG8 (1:1000 for WB, 12661-1-AP, Proteintech), anti-Syntaxin 5 (1:3000 for WB, 110053, Synaptic Systems), anti-Syntaxin 6 (1:1000 for WB, 110062, Synaptic Systems), anti-GS27 (1:1000 for WB, 12095-1-AP, Proteintech), anti-GAPDH (1:5000 for WB, KC-5G5, Kangchen Bio-tech), HRP conjugated anti-His-tag (1:5000 for WB, HRP66005, Proteintech), anti-poly(ADP-ribose) (1:200 for IP, 1020/N, Tulip Biolabs), anti-SNAP tag (1:1000 for WB, P9310S, NEB), anti-collagen IV (1:300 for IF, ab6586, Abcam), HRP conjugated Wheat Germ Agglutinin (1:5000 for WB, WGA-HRP, 29073, biotium). Anti-Rabbit Alexa Fluor 488 (1:500 for IF, A21441), Alexa Fluor 568 (1:500 for IF, A10042), Alexa Fluor 647 (1:500 for IF, A21245) and anti-Mouse Alexa Fluor 488 (1:500 for IF, A21200), Alexa Fluor 568 (1:500 for IF, A10037), Alexa Fluor 647 (1:500 for IF, A21236) for Immunofluorescence were obtained from ThermoFisher.
Validation	Commercial antibodies have been verified by the manufacturers as shown on their website. All antibodies were used based on manufacturer recommendations.

Eukaryotic cell lines

Policy information about cell lines

Cell line source(s)	HeLa cell line was purchased from the American Type Culture Collection. Cos7 cell line was kindly provided by Stem Cell Bank,

	Chinese Academy of Sciences.
Authentication	HeLa cells were authenticated by STR profiling. The authentication of Cos7 is provided by Stem Cell Bank, Chinese Academy of Sciences.
Mycoplasma contamination	All cell lines were routinely tested for the mycoplasma contamination and were negative.
Commonly misidentified lines (See ICLAC register)	None of the cell lines used in this study is listed in the database of commonly misidentified cell kines maintained by iCLAC.
